# Integrated GC–MS and LC–MS metabolomics reveals grade-dependent chemical variation and antioxidant activity of Gougunao tea

**DOI:** 10.1016/j.fochx.2026.104721

**Published:** 2026-10-29

**Authors:** Fengke Lin, Qun Zhou, Nuo Chen, Ying Ren, Wenkai Zhao, Jianbo Nie, Yixin Qin, Trevor N. Clark, Chunsong Cheng, Binsheng Luo

**Affiliations:** a School of Chemistry and Chemical Engineering, Tianjin University of Technology, Tianjin 300384, China; b Jiangxi Key Laboratory for Sustainable Utilization of Chinese Materia Medica Resources, Lushan Botanical Garden, Chinese Academy of Sciences, Jiujiang 332900, Jiangxi Province, China; c Lushan Xinglin Institute for Medicinal Plants, Jiujiang Xinglin Key Laboratory for Traditional Chinese Medicines, Jiujiang 332900, Jiangxi Province, China; d Department of Chemistry, University of Prince Edward Island, Charlottetown, PE C1A 4P3, Canada

**Keywords:** Gougunao tea, Green tea, GC–MS, LC–MS, Metabolomics, Chemometrics, Grade differentiation, Antioxidant activity

## Abstract

Gougunao tea (GT), a renowned Chinese green tea, is commercially classified into multiple quality grades, yet the metabolomic basis of grade differentiation and antioxidant potential remains unclear. In the current study, integrated HS-SPME-GC–MS and UPLC-QE-Orbitrap-HRMS metabolomics, chemometrics, and antioxidant assays were used to characterize six commercial GT grades (G1-G6). In total, 137 volatile compounds and 328 non-volatile features were annotated. Alcohols, aldehydes, and ketones dominated the volatile profiles, whereas fatty acid derivatives, flavonoids, amino acids, and peptides were major non-volatile classes. PCA and PLS-DA revealed grade-associated metabolic differences, with 81 features (31 volatile and 50 non-volatile) screened as candidate grade-associated features. GT samples showed strong DPPH, ABTS, and FRAP antioxidant activities, with G6 generally showing better antioxidant performance. Correlation analysis indicated that several flavonoids were positively associated with antioxidant capacity. These findings provide metabolomic evidence for GT grade differentiation and identify candidate chemical features associated with commercial grade.

## Highlights

• Integrated GC–MS/LC–MS metabolomics profiled six Gougunao tea grades.
• Chemometric models clearly discriminated Gougunao tea grades.
• A total of 81 features were screened as candidate grade-associated markers.
• Gougunao tea showed strong DPPH, ABTS, and FRAP antioxidant activities.
• Flavonoids were associated with the antioxidant potential of Gougunao tea.

## 1 Introduction

Tea produced from *Camellia sinensis* represents one of the most widely consumed beverages worldwide and holds substantial cultural, social, and economic importance (Sang, Lambert, Ho, & Yang, 2011). The sensory quality of tea is largely determined by a complex mixture of volatile and non-volatile constituents formed during plant growth, harvesting, and post-harvest processing (Ho, Zheng, & Li, 2015). Beyond aroma and taste, tea has attracted considerable scientific interest for its diverse biological activities, including antioxidant, anti-inflammatory, and cardioprotective effects, which are closely associated with health-promoting potential (Khan & Mukhtar, 2013). Therefore, chemical characterization combined with bioactivity evaluation has become an important strategy for understanding tea quality, authenticity, and functional value (Zhang et al., 2019).

China produces a wide variety of teas, including green, black, and dark teas (Pan et al., 2022). Among them, Gougunao tea (GT) is a renowned regional green tea with a consumption history of more than 200 years. The name “Gougunao” originates from Gougunao Mountain in Suichuan County, Jiangxi Province, where favorable ecological conditions support the production of high-quality tea (Guo, 1994). GT has received numerous regional and national recognitions, including Geographical Indication Product status and China Time-honored Brand-related recognition, highlighting its cultural, historical, and commercial significance (Xiao, 2017). According to the Chinese National Standard GB/T 19691–2008 (General Administration of Quality Supervision, Inspection and Quarantine of the People's Republic of China, & Standardization Administration of China, 2008), GT is classified into six commercial quality grades, namely Premium Special Grade, Tribute Special Grade, Supreme Special Grade, Special Grade, Grade I, and Standard Grade, in descending order of commercial quality. Previous studies have shown that volatile and non-volatile metabolites differ substantially among different grades of green tea and oolong tea (Xia et al., 2026; Zeng et al., 2023), indicating that metabolomic profiling can provide valuable chemical evidence for tea grade discrimination and quality evaluation. Nevertheless, the underlying chemical differences among GT grade samples remain unexamined.

Recent advances in headspace solid-phase microextraction coupled with gas chromatography–mass spectrometry (HS-SPME-GC–MS) have enabled efficient, solvent-free, and sensitive profiling of volatile compounds in tea and other plant materials (Wang et al., 2022). In parallel, ultra-performance liquid chromatography coupled with high-resolution mass spectrometry (UPLC-HRMS), such as UPLC-QE-Orbitrap-HRMS, provides high sensitivity and accurate mass measurement for the comprehensive analysis of non-volatile constituents, including flavonoids, phenolic acids, alkaloids, amino acids, and other secondary metabolites (Ling et al., 2022). The integration of GC–MS- and LC–MS-based metabolomics offers complementary coverage of the tea metabolome and enables a more comprehensive understanding of chemical variation (de Souza et al., 2016). When combined with chemometric methods such as principal component analysis (PCA) and partial least squares discriminant analysis (PLS-DA), metabolomics has become a powerful approach for sample discrimination, biomarker discovery, and authenticity assessment (Lin et al., 2025; Xia et al., 2026). Although an integrated GC–MS and LC–MS metabolomics approach has been applied to discriminate GT samples from different geographical origins (Sun et al., 2025), grade-dependent chemical variation in GT has not been investigated using this strategy.

In addition to chemical profiling, evaluating the functional relevance of metabolite variation is essential for understanding tea quality from a health-related perspective (Cabrera, Artacho, & Giménez, 2006; Vuong, 2014). Antioxidant capacity, commonly assessed by 2,2-diphenyl-1-picrylhydrazyl (DPPH), 2,2′-azinobis-(3-ethylbenzothiazoline-6-sulfonic acid) (ABTS), and ferric reducing antioxidant power (FRAP) assays, is widely used as an important indicator of the functional potential of tea (Gorjanović et al., 2012). However, the relationship between grade-dependent metabolomic variation and antioxidant capacity has not been studied in GT. Clarifying this relationship would provide useful information for evaluating both the quality and functional value of GT products.

In this study, an integrated HS-SPME-GC–MS and UPLC-QE-Orbitrap-HRMS metabolomics strategy combined with chemometric analysis was employed to characterize volatile and non-volatile metabolite differences among six commercial grades of GT. The antioxidant activities of GT samples were evaluated using DPPH, ABTS, and FRAP assays, and correlation analysis was further performed to identify metabolites potentially associated with antioxidant capacity. This work provides a comprehensive characterization of the metabolomic profiles of GT across commercial grades and identifies candidate chemical features for further evaluation of their associations with commercial grade and antioxidant activity.

## 2 Materials and methods

### 2.1 Chemicals and reagents

Analytical-grade methanol used for tea sample extraction was purchased from Concord Technology Co., Ltd. (Tianjin, China). For HS-SPME-GC–MS analysis, 2-octanol, used as the internal standard, was obtained from Tokyo Chemical Industry Co., Ltd. (Tokyo, Japan), and a homologous series of *n*-alkane standards (C7-C30) was supplied by Anpel Laboratory Technologies Inc. (Shanghai, China). For UPLC-QE-Orbitrap-HRMS analysis, LC-MS-grade methanol, acetonitrile, and isopropanol were purchased from CNW Technologies GmbH (Düsseldorf, Germany), whereas LC-MS-grade acetic acid was obtained from Sigma-Aldrich (St. Louis, MO, USA). Ultrapure water was obtained from Watsons (Hong Kong, China). The internal standards 2-chloro-*L*-phenylalanine and decanoic acid‑*d*_19_ were purchased from Zhenzhun Biotechnology Co., Ltd. (Shanghai, China) and Manhage Biotechnology Co., Ltd. (Shanghai, China), respectively. For antioxidant assays, analytical-grade dimethyl sulfoxide (DMSO) was supplied by Concord Technology Co., Ltd. DPPH, ABTS, ferric chloride hexahydrate (FeCl_3_·6H_2_O), ferrous sulfate monohydrate (FeSO_4_·H_2_O), 2,4,6-tris(2-pyridyl)-*s*-triazine (TPTZ), analytical-grade methanol, and ethanol were purchased from Macklin Biochemical Co., Ltd. (Shanghai, China). Acetate buffer solution (300 mM, pH 3.6) was obtained from Shanghai Yuanye Biotechnology Co., Ltd. (Shanghai, China). l-Ascorbic acid (LAA) and potassium persulfate (K_2_S_2_O_8_) were supplied by Aladdin Biochemical Technology Co., Ltd. (Shanghai, China).

### 2.2 Tea material collection and preparation

Six commercial grades of GT, namely Premium Special Grade (G1), Tribute Special Grade (G2), Supreme Special Grade (G3), Special Grade (G4), Grade I (G5), and Standard Grade (G6), were obtained from Jiangxi Honglu Tea Industry Co., Ltd. (Suichuan County, Jiangxi Province, China). These grades represent a descending order of commercial quality and are mainly distinguished by plucking standard, leaf tenderness and sensory quality. All samples were harvested from the same plantation in Suichuan County in 2024 to reduce geographical variation. Tea material collection, processing, and grade classification were performed according to the Chinese National Standard GB/T 19691–2008, jointly issued by the Standardization Administration of the People's Republic of China and the General Administration of Quality Supervision, Inspection and Quarantine of the People's Republic of China. For each grade, samples were collected from three independent production batches, with three individual samples obtained from each batch, resulting in nine individual samples per grade. After collection, the samples were sealed, transported to the laboratory, and stored at −80 °C until sample preparation. To obtain a representative sample for each grade and reduce individual sample variation, equal amounts of the nine samples were pooled and thoroughly homogenized to generate one grade-level composite sample for subsequent analysis.

### 2.3 HS-SPME-GC–MS analysis

Tea samples were ground into fine powder under liquid nitrogen. For each grade, five analytical replicates were prepared by independently weighing approximately 800 mg of the homogenized grade-level composite sample into separate 20 mL headspace vials. Each vial was then supplemented with 10 μL of 2-octanol solution (10 μg/mL in deionized water) as the internal standard and immediately sealed. 2-Octanol was used to monitor analytical stability and repeatability. A blank run was performed prior to sample analysis to monitor background contamination and system cleanliness. After equilibration at 60 °C for 15 min, volatile compounds were extracted from the headspace for 30 min using a 50/30 μm DVB/CAR/PDMS StableFlex fiber (2 cm, Supelco, Bellefonte, PA, USA), followed by thermal desorption in the GC injector for 4 min. GC–MS analysis was performed using an Agilent 7890 gas chromatograph coupled to an Agilent 5977B mass spectrometer (Agilent Technologies, Santa Clara, CA, USA). Chromatographic separation was achieved on a DB-Wax capillary column (30 m × 0.25 mm × 0.25 μm, Agilent Technologies). Helium was used as the carrier gas at a constant flow rate of 1.0 mL/min. Samples were injected in splitless mode. The oven temperature was initially maintained at 40 °C for 4 min, increased to 245 °C at 5 °C/min, and then held at 245 °C for 5 min. The injector, transfer line, ion source, and quadrupole temperatures were set at 250, 250, 230, and 150 °C, respectively. Mass spectra were acquired in electron impact ionization mode at 70 eV over an *m*/*z* range of 20–400. Raw GC–MS data were processed using ChromaTOF 4.3× software (LECO Corporation, St. Joseph, MI, USA) for peak detection, baseline correction, deconvolution, alignment, and peak area integration. Linear retention indices (LRIs) were calculated using a homologous series of *n*-alkane standards (C_7_-C_30_) analyzed under the same chromatographic conditions. Identification of volatile compounds was performed by comparing the obtained mass spectra with those in the NIST 23 mass spectral library, together with LRI matching against reference values reported in the NIST Chemistry WebBook (https://webbook.nist.gov/chemistry/) for DB-Wax columns. To ensure the reliability of compound identification, only annotations with a mass spectral similarity score > 700 and a RI deviation within ±30 were retained (Li et al., 2025). Representative odor descriptions of the identified volatile compounds were retrieved from publicly accessible databases, including Flavornet (https://www.flavornet.org/), FooDB (https://foodb.ca/), and FlavorDB2 (https://cosylab.iiitd.edu.in/flavordb2/). The relative abundance of each volatile compound was expressed as the percentage of its peak area relative to the total ion chromatogram.

### 2.4 UPLC-QE-Orbitrap-HRMS analysis

For each GT grade, five analytical extraction replicates were prepared by separately weighing five 2.0 g aliquots of powdered tea from the same homogenized grade-level composite sample. Each aliquot was extracted with 35 mL of 75% methanol (*v*/*v*) by ultrasonic-assisted extraction for 30 min. For each replicate, the extraction was repeated three times, and the three resulting supernatants were combined separately, filtered, and concentrated under reduced pressure using an RE-2000 rotary evaporator (Tianjin Kenuo Instrument Equipment Co., Ltd., Tianjin, China). The residual solvent was removed by freeze-drying for 48 h using an LGJ-10C lyophilizer (Sihuan Forui Technology Development Co., Ltd., Beijing, China). This procedure yielded five independently prepared dried extracts for each grade. Each dried extract was individually reconstituted in 75% methanol to a concentration of 1 mg/mL for LC–MS analysis. Internal standards, including 2-chloro-*L*-phenylalanine (2.0 μM) and decanoic acid‑*d*_19_ (0.16 μM), were added to each sample solution. The internal standards were used to monitor analytical stability and repeatability. A pooled quality control (QC) sample was prepared by mixing equal aliquots of all reconstituted sample solutions. Chromatographic separation was performed on a Vanquish UHPLC system (Thermo Fisher Scientific, Waltham, MA, USA) equipped with a Phenomenex Kinetex C18 column (2.1 × 100 mm, 2.6 μm). The column temperature was maintained at 25 °C, and the autosampler temperature was set at 4 °C. The mobile phase consisted of water containing 0.01% acetic acid (A) and isopropanol/acetonitrile (1:1, *v*/*v*) (B). The flow rate was 0.3 mL/min, and the injection volume was 1 μL. The gradient elution program was as follows: 0–1.0 min, 1% B; 1.0–8.0 min, 1–99% B; 8.0–9.0, 99% B; 9.0–9.1 min, 99–1% B; and 9.1–12.0 min, 1% B. Mass spectrometric analysis was carried out using an Orbitrap Exploris 120 mass spectrometer (Thermo Fisher Scientific) equipped with an electrospray ionization source and operated in both positive and negative ion modes. MS/MS data were acquired using data-dependent acquisition. The main parameters were as follows: sheath gas, 50 arb; auxiliary gas, 15 arb; sweep gas, 1 arb; capillary temperature, 320 °C; vaporizer temperature, 350 °C; spray voltage, +3.8 kV in positive ion mode and − 3.4 kV in negative ion mode; full-scan resolution, 60,000; MS/MS resolution, 15,000; and stepped collision energies of 20, 30, and 40 eV. Raw data files were converted to mzXML format using ProteoWizard and then processed by XCMS for peak detection, retention time correction, alignment, and peak area integration. Blank-related features were excluded before subsequent metabolite annotation and statistical analysis.

Metabolite annotation was performed against the in-house BiotreeDB database (version 3.0), which was established using authentic reference standards analyzed on the same LC–MS platform and under identical chromatographic and mass-spectrometric conditions as those used for the GT samples. Sample features were matched to reference standards based on accurate precursor *m/z*, retention time (RT), and MS/MS fragmentation spectra. Candidate matches were evaluated using the previously reported MetDNA2 scoring strategy (Zhou et al., 2022), according to the following equation: *S*_total_ = *W*_*m/z*_ × *S*_*m/z*_ + *W*_RT_ × *S*_RT_ + *W*_MS/MS_ × *S*_MS/MS_ + *S*_confidence_, where *S*_*m/z*_, *S*_RT_, and *S*_MS/MS_ represent the *m*/*z*, RT, and MS/MS match scores, respectively. The corresponding weighting factors (*W*_*m/z*_, *W*_RT_ and *W*_MS/MS_) were 0.25, 0.25, and 0.50, respectively (Zhou et al., 2022). The *m/z* and RT match scores were calculated according to the scoring functions described in MetDNA (Shen et al., 2019), whereas MS/MS spectral similarity was evaluated using the square-root-transformed dot-product score (Li et al., 2021). For MSI Level 1 identification, the confidence score was assigned a value of 3, giving a maximum total score of 4.0. To ensure stringent metabolite identification in the present study, only candidate matches satisfying all of the following criteria were retained: *S*_total_ ≥ 3.6, *S*_*m/z*_ ≥ 0.667 (corresponding to a precursor mass error ≤ 5 ppm), *S*_RT_ ≥ 0.50 (corresponding to an RT deviation ≤10 s), and MS/MS match score ≥ 0.80. According to the MSI criteria (Blaženović et al., 2019; Sumner et al., 2007), features showing a unique match within the available authentic-standard library based on RT, accurate precursor *m/z*, and MS/MS spectra were classified as MSI Level 1 identifications. When two or more isomeric reference standards could not be unambiguously distinguished under the present chromatographic and MS/MS conditions, the corresponding signal was conservatively reported as an unresolved isomeric feature rather than assigned to an individual isomer.

### 2.5 Antioxidant evaluations

The antioxidant capacities of GT samples from different grades were evaluated using three complementary in vitro assays, including DPPH radical-scavenging, ABTS radical-scavenging, and ferric reducing antioxidant power (FRAP) assays. For each grade, 10 mg was weighed from each of the five independently prepared dried extracts used for LC–MS metabolomic analysis, and these aliquots were combined to obtain a 50 mg pooled extract for subsequent antioxidant assays. The pooled extract was dissolved in DMSO to prepare a stock solution of 50 mg/mL and stored at −20 °C until subsequent antioxidant analysis.

#### 2.5.1 DPPH radical-scavenging assay

The DPPH radical-scavenging activity was determined using a previously reported method with minor modifications (Awasthi et al., 2025). Briefly, freshly prepared DPPH solution (0.1 mM in methanol) was used for the assay. A 500 μg/mL working solution of each grade extract was prepared by appropriate dilution of the 50 mg/mL stock solution. In each well of a 96-well microplate, 20 μL of the working solution was mixed with 180 μL of DPPH solution to obtain a final extract concentration of 50 μg/mL. The reaction mixture was incubated at 37 °C in the dark for 30 min. LAA at the same final concentration was used as the positive control, and DMSO was used as the negative control. The absorbance was measured at 517 nm using an Epoch microplate spectrophotometer (BioTek Instruments Inc., Winooski, VT, USA). The DPPH radical-scavenging activity was calculated as follows: inhibition (%) = [1 − (A_sample/A_control)] × 100, where A_sample and A_control represent the absorbance values of the sample-treated group and the negative control group, respectively. Samples showing more than 50% inhibition were further diluted to determine their IC_50_ values.

#### 2.5.2 ABTS radical-scavenging assay

The ABTS radical-scavenging activity was evaluated using the ABTS•^+^ decolorization assay. The ABTS•^+^ stock solution was prepared by mixing 7 mM ABTS solution with 2.45 mM potassium persulfate and allowing the mixture to react in the dark at room temperature for 12–16 h. Before use, the ABTS•^+^ solution was diluted with ethanol to an absorbance of 0.70 ± 0.02 at 734 nm. A 500 μg/mL working solution of each grade extract was prepared by appropriate dilution of the 50 mg/mL stock solution. For the assay, 20 μL of sample solution was mixed with 180 μL of diluted ABTS•^+^ solution in a 96-well plate to obtain a final sample concentration of 50 μg/mL. The reaction was performed at 37 °C in the dark for 30 min. LAA at the same final concentration was used as the positive control, and DMSO was used as the negative control. The absorbance was recorded at 734 nm using an Epoch microplate spectrophotometer. The ABTS radical-scavenging activity was calculated as follows: inhibition (%) = [1 − (A_sample/A_control)] × 100, where A_sample and A_control denote the absorbance values of the sample-treated group and the negative control group, respectively. Samples with inhibition rates greater than 50% were serially diluted for IC_50_ determination.

#### 2.5.3 FRAP assay

The ferric reducing antioxidant power was measured using the FRAP assay. The FRAP working reagent was freshly prepared by mixing acetate buffer (300 mM, pH 3.6), TPTZ solution (10 mM in 40 mM HCl), and FeCl_3_ solution (20 mM) at a volume ratio of 10:1:1. Sample working solutions at concentrations of 31.3, 62.5, and 125 μg/mL were prepared from the stock solution. In each well of a 96-well plate, 20 μL of the sample working solution was mixed with 180 μL of FRAP working reagent, giving final extract concentrations of 3.13, 6.25, and 12.5 μg/mL, respectively. The mixture was incubated at 37 °C in the dark for 30 min. LAA was used as the positive control, and DMSO was used as the negative control. The absorbance was measured at 595 nm. The reducing capacity was calculated using a calibration curve prepared with FeSO_4_ standard solutions at final concentrations ranging from 6.25 to 100 μM. The results were expressed as μmol Fe^2+^ equivalents/L.

### 2.6 Statistical analysis

HS-SPME-GC–MS and UPLC-QE-Orbitrap-HRMS analyses were performed in five analytical replicates for each grade. Multivariate statistical analyses, including PCA and PLS-DA, were performed using SIMCA-P 14.1 software (Umetrics, Umeå, Sweden). Univariate comparisons among GT grades were conducted using one-way analysis of variance (ANOVA) in IBM SPSS Statistics 27 (IBM Corp., Armonk, NY, USA). Permutation tests (with 200 random permutations) were performed to evaluate the possibility of overfitting. Grade-associated candidate features were selected according to the combined criteria of variable importance in projection (VIP) values >2.0 in the PLS-DA models and ANOVA *p*-values <0.05. Bioactivity data were obtained from three independent measurements and are expressed as mean ± standard deviation. Differences among all GT sample groups were further assessed using Duncan's multiple range test after one-way ANOVA, and *p* < 0.05 was considered statistically significant. Spearman correlation analysis was conducted across the six GT grades (*n* = 6) using the mean relative abundance of each grade-associated candidate metabolite and the corresponding mean antioxidant value for each grade. Correlation coefficients and two-tailed *p*-values were calculated using IBM SPSS Statistics 27. Correlation matrices were visualized using GraphPad Prism 10.5.0 (GraphPad Software LLC, Boston, MA, USA).

## 3 Results and discussion

### 3.1 Volatile profiles of GT samples based on HS-SPME-GC–MS metabolomics

The volatile profiles of GT samples from six commercial grades were detected using HS-SPME-GC–MS-based untargeted metabolomics. A total of 137 volatile compounds were characterized and grouped into 17 chemical classes, including alcohols, aldehydes, ketones, esters, monoterpenes, and nitrogen-containing heterocycles (Table S1). This result indicates the high chemical complexity of the GT volatilome. The number of volatile compounds detected in this study was higher than that reported for several other green teas, including Yichang big-leaf green teas of different grades, for which 94 volatile compounds were identified (Yin et al., 2022). Marked differences in the relative abundances of volatile classes were observed among the six GT grades (Fig. 1). Alcohols were the most abundant class, accounting for 25.45–33.72% of the total volatiles, followed by aldehydes (17.91–21.45%), ketones (6.70–9.50%), monoterpenes (2.05–9.05%), and aromatic hydrocarbons (2.21–2.80%) (Fig. 1). A similar predominance of alcohols, aldehydes, and ketones has been reported in other green teas, including Fuliang green tea and Longjing green tea (Li et al., 2024; Xu et al., 2024; Zhang et al., 2024), suggesting that these classes represent important components of the volatile matrix of green tea. Among the individual compounds, 1-penten-3-ol was the most abundant volatile constituent, with relative contents ranging from 8.38% to 20.10%, followed by pentanal (11.70–15.33%), benzeneethanol (2.74–4.64%), pentanol (2.07–3.23%), and *cis*-2-pentenol (1.68–3.24%) (Table S1). These compounds have also been detected in various tea products, including Longjing green tea and pan-fired green teas (Kato & Shibamoto, 2001; Lee, Chambers IV, E., Adhikari, & Yoon, 2013; Lin et al., 2024; Zhang et al., 2024), indicating their potential contribution to characteristic green tea aroma. Several volatile compounds identified in this study, such as linalool, olivetol, and *cis*-3-hexenyl hexanoate, have previously been reported in GT samples from different geographical origins (Sun et al., 2025). However, some major constituents detected in the present study, including pentanal, 1-penten-3-ol, and benzeneethanol, were not included in previous GT studies. These differences may be related to variations in geographical origin, harvesting season, sample preparation, and analytical methods (Zeng, Watanabe, & Yang, 2019). Among the identified volatile compounds, 89 compounds (64.96%) were associated with reported odor descriptors (Table S1). For example, 1-penten-3-ol is generally described as having buttery, pungent, and green notes, whereas pentanal is associated with almond, malt, and pungent odors. Abundance variation in these aroma-related volatiles may partly contribute to the grade-dependent differences in GT volatiles. However, their actual sensory contributions require further confirmation by sensory-directed approaches such as GC-O analysis.

**Fig. 1:**
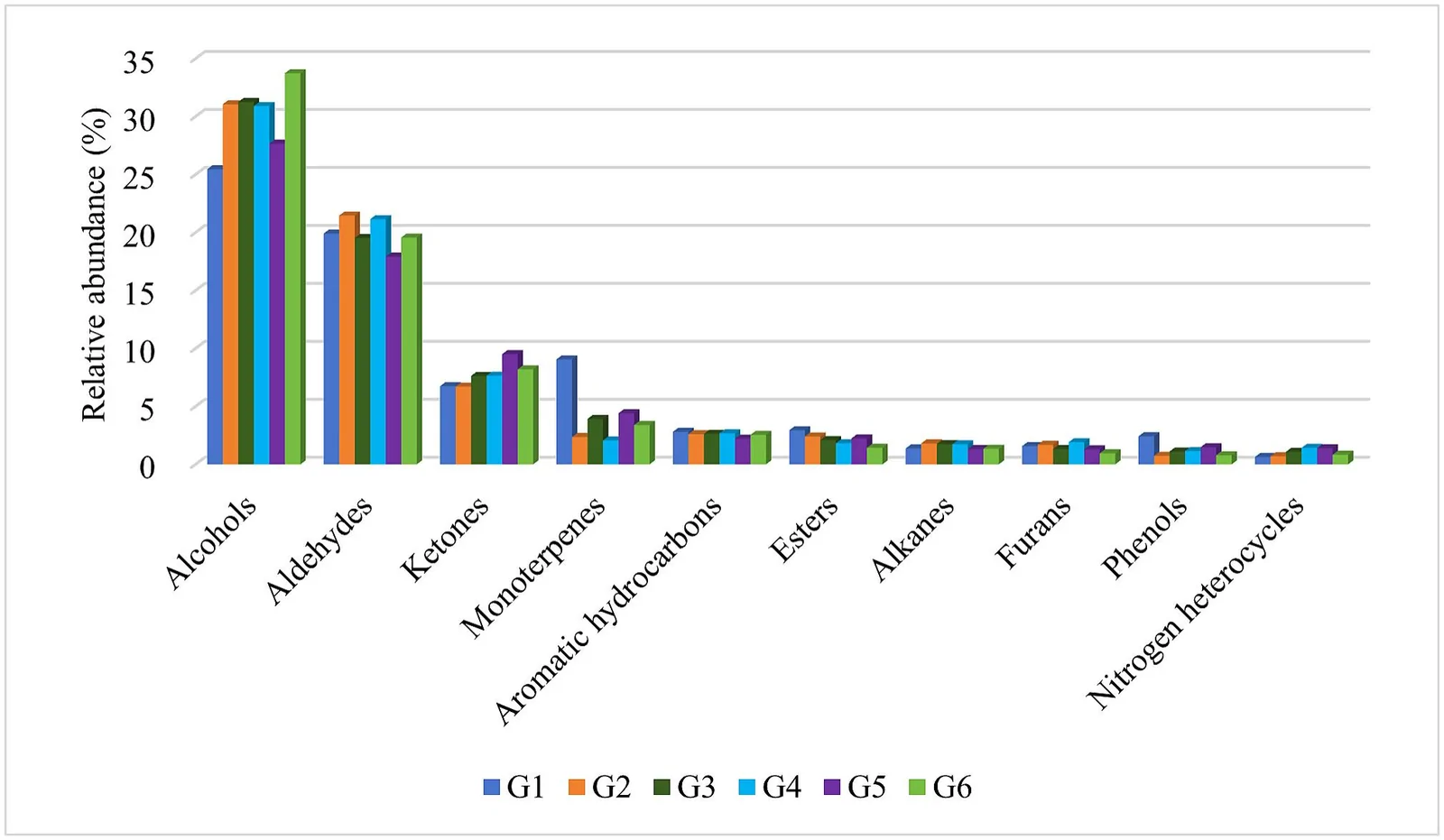
Relative abundances of the ten major volatile chemical classes detected in GT samples across six quality grades by HS-SPME-GC–MS. G1-G6 represent Premium Special Grade, Tribute Special Grade, Supreme Special Grade, Special Grade, Grade I, and Standard Grade, respectively.

### 3.2 Non-volatile profiles of GT samples based on UPLC-QE-Orbitrap-HRMS metabolomics

The non-volatile metabolite profiles of GT samples from different grades were characterized using untargeted metabolomics based on UPLC-QE-Orbitrap-HRMS. A total of 4388 unique analytical features had DDA-acquired MS/MS information, including 2731 in positive ion mode and 1657 in negative ion mode, whereas 51,308 features had only MS1-level annotations without corresponding MS/MS evidence. After further filtering according to the predefined identification criteria, 328 analytical features were annotated, including 288 MSI Level 1 identifications and 40 unresolved isomeric features. Among these features, 159 were detected in positive ion mode and 225 in negative ion mode, with 56 detected in both modes (Table S2). The MSI Level 1 assignments were based on unique matches within the available authentic-standard library using RT, accurate precursor m/z, and MS/MS spectra obtained under identical chromatographic and mass-spectrometric conditions, and therefore represent a high-confidence category of metabolite identification (Blaženović et al., 2019; Sumner et al., 2007). However, features that could not be unambiguously distinguished among two or more isomeric reference standards were reported as unresolved isomeric features rather than assigned to a single structure. The in-house reference-standard library was established to provide broad metabolite coverage and was not restricted to compounds previously reported in *Camellia* species. Therefore, taxonomic occurrence was not used as a prior exclusion criterion. Several retained annotations, including 6-hydroxy-4-methylcoumarin and 8-geranyl-7-hydroxycoumarin, have not been commonly reported in green tea. Although these annotations satisfied the predefined matching criteria against the available authentic-standard library, their occurrence in green tea should be interpreted cautiously. Additional orthogonal evidence, such as isolation followed by NMR characterization, could provide further support for the structural assignments of these unusual metabolites.

The characterized features were assigned to 20 chemical classes, including fatty acids and their derivatives (70 features), flavonoids (42), amino acids and peptides (37), glycerophospholipids (33), and nucleotides and derivatives (23) (Fig. 2). These findings demonstrate the considerable chemical diversity of the non-volatile metabolome of GT. The major metabolite classes identified here provide a broad chemical basis for examining metabolic variation among commercial grades. Differences in their abundance may contribute to the grade-associated separation observed in the subsequent multivariate analyses. The presence of nutritional components such as amino acids and peptides, together with flavonoids and phenylpropanoids with reported bioactivities, also suggests the nutritional and functional relevance of GT (Heleno, Martins, Queiroz, & Ferreira, 2015; Williamson, Kay, & Crozier, 2018). Altogether, the present study systematically characterized non-volatile metabolites in GT samples across commercial grades using UPLC-QE-Orbitrap-HRMS-based untargeted metabolomics, providing a basis for the subsequent identification of grade-associated candidate features and candidate metabolites associated with antioxidant activity.

**Fig. 2:**
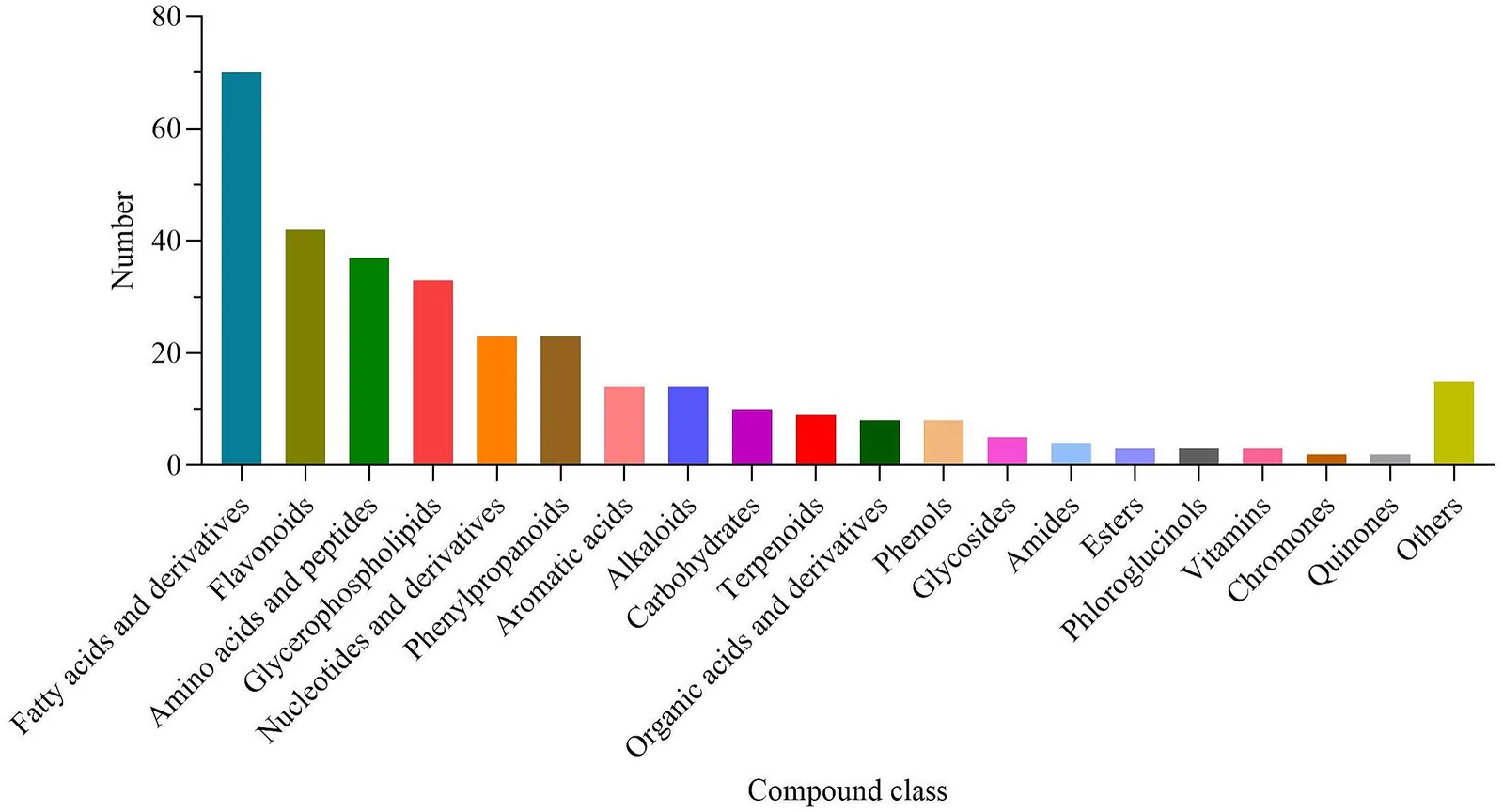
Classification of non-volatile features detected in GT samples and the number of compounds in each chemical class.

### 3.3 Grade-dependent chemical variation of GT revealed by chemometric analysis

To evaluate metabolic differences across GT grades, PCA and PLS-DA were performed on the volatile and non-volatile metabolomic datasets. Three PCA models (Fig. 3A-C) and three corresponding PLS-DA models (Fig. 3D-F) were constructed from HS-SPME-GC–MS and UPLC-QE-Orbitrap-HRMS data acquired in positive and negative ion modes. PCA was used to examine the overall variation in the metabolomic datasets, whereas PLS-DA was employed as an exploratory supervised analysis to further examine grade-associated patterns within the present dataset. In the PCA models, the first two principal components explained more than 50% of the total variance in all datasets, indicating that the main metabolic variation among GT samples was effectively represented (Fig. 3A-C). The PLS-DA models showed clear separation patterns within the present analytical dataset, with R^2^X(cum), R^2^Y(cum), and Q^2^(cum) values all exceeding 0.83 (Table S3). Moreover, permutation tests showed negative Q^2^ intercepts, suggesting that the PLS-DA models were not overfitted within the present dataset (Fig. 3D1-F1) (Westerhuis et al., 2008). Given that the replicate measurements were analytical replicates derived from grade-level composite samples rather than independent biological replicates, the PLS-DA models were interpreted as exploratory pattern-recognition models rather than independently validated classification models.

**Fig. 3:**
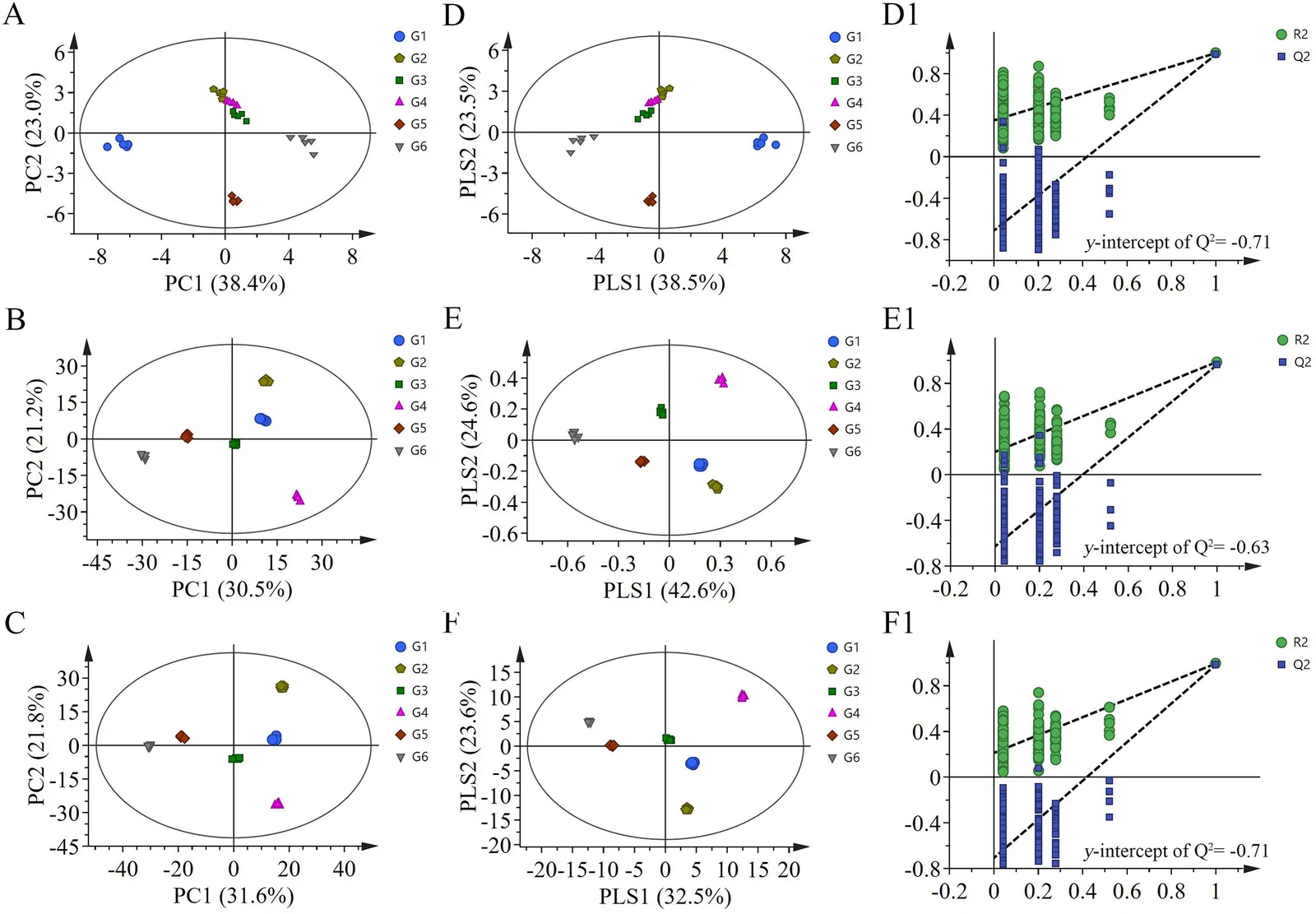
Chemometric analysis of GT samples across six quality grades. (A-C) PCA score plots based on HS-SPME-GC–MS, UPLC-QE-Orbitrap-HRMS positive ion mode, and UPLC-QE-Orbitrap-HRMS negative ion mode datasets, respectively. (D-F) Corresponding PLS-DA score plots. (D1-F1) Permutation tests of the PLS-DA models shown in D-F. G1-G6 represent Premium Special Grade, Tribute Special Grade, Supreme Special Grade, Special Grade, Grade I, and Standard Grade, respectively. HS-SPME-GC–MS and UPLC-QE-Orbitrap-HRMS analyses were conducted in five analytical replicates for each grade.

For volatile metabolites, both PCA and PLS-DA score plots showed that G4 clustered closely with G2 and G3, whereas the other grades were more clearly separated (Fig. 3A and D). This result suggests that the volatile profiles of the intermediate grades were relatively similar, while distinct volatile characteristics were observed among higher- and lower-grade samples. Similar grade-dependent variations in volatile profiles have also been reported for other green teas, such as Yichang big-leaf green tea (Yin et al., 2022). Compared with the GC–MS dataset, the LC–MS datasets showed more pronounced grade-associated separation patterns. In both positive and negative ion modes, all six GT grades formed distinct clusters in the PCA and exploratory PLS-DA score plots (Fig. 3B, C, E, and F), indicating that non-volatile metabolites provided substantial chemical information associated with commercial grade within the present sample set. According to GB/T 19691–2008, different GT grades have different requirements for plucking standards, with higher grades generally requiring younger and more tender shoots and lower grades permitting more developed leaf materials. In contrast, the same general processing sequence is prescribed across the six grades. Thus, differences in plucking standard, leaf developmental stage, and tenderness may be important factors underlying the observed metabolic variation. The standard also specifies grade-dependent sensory characteristics, including aroma and taste, suggesting that the observed chemical differences may contribute to the sensory characteristics associated with commercial grading. Taken together, the grade-associated chemical separation may partly reflect differences in raw-leaf maturity and tenderness among the commercial grades. Previous integrated GC–MS and LC–MS metabolomic studies of GT have mainly focused on geographical-origin discrimination (Sun et al., 2025), whereas the present study extends this integrated approach to commercial-grade differentiation and provides complementary chemical information on grade-associated variation in GT.

### 3.4 Preliminary grade-associated candidate metabolites in GT

To further clarify the chemical basis underlying the grade-dependent separation observed in the multivariate analyses, candidate metabolites were preliminarily screened from both volatile and non-volatile datasets using the combined criteria of VIP > 2.0 in the exploratory PLS-DA models and ANOVA *p* < 0.05 (Liang et al., 2025; Lin & Long, 2023). Because the statistical analyses were based on analytical replicates prepared from grade-level composite samples, these *p*-values were used for exploratory screening rather than population-level inference. Accordingly, the screened metabolites are referred to here as preliminary grade-associated candidate features. In total, 81 grade-associated candidate metabolites were obtained, including 31 volatile compounds from the HS-SPME-GC–MS dataset and 50 non-volatile metabolite annotations from the LC–MS datasets. The candidate volatile compounds mainly included alcohols, ketones, and monoterpenes (Table 1). For the LC–MS datasets, 21 and 29 candidate feature entries were obtained in positive and negative ion modes, respectively, including flavonoids, alkaloids, and amino acids and peptides (Table 2). Among the candidate volatile compounds, 1-penten-3-ol showed the highest VIP value (VIP = 6.74), followed by cyclofenchene (5.84), pentanal (5.44), benzeneethanol (4.43), and linalool (4.21) (Table 1), indicating their relatively strong contributions to the grade-associated separation observed in the exploratory PLS-DA model. In the positive ion mode LC–MS dataset, caffeine (9.77), theanine (7.53), linoleamide (6.02), theophylline (5.81), and epigallocatechin (4.31) were among the major contributors to the observed separation pattern. In the negative ion mode dataset, 4-*p*-coumaroylquinic acid (6.64), procyanidin B2 (5.75), 3-(2,4-dihydroxypentyl)-6,8-dihydroxyisochromen-1-one (5.74), epigallocatechin (5.32), and leucocyanidin (5.32) showed the highest VIP values (Table 2). Notably, seven metabolites, such as theanine, epigallocatechin, and (+)-gallocatechin, were prioritized in both ionization modes, further supporting their potential association with grade-related chemical variation within the present dataset.

**Table 1:** Preliminary grade-associated volatile candidate features identified by exploratory screening.

No.^a^	Compound	LRI^b^	Class	VIP value^c^	*p* value^d^
1	1-Methoxypropan-2-ol	1125	Alcohols	2.28	<0.001
3	1-Penten-3-ol	1161	Alcohols	6.74	<0.001
4	Pentanol	1252	Alcohols	3.33	<0.001
6	*cis*-2-Pentenol	1320	Alcohols	2.84	<0.001
9	*cis*-3-Hexene-1-ol	1384	Alcohols	3.07	<0.001
12	Morillol	1452	Alcohols	2.56	<0.001
17	Phenylmethanol	1872	Alcohols	4.12	<0.001
18	Benzeneethanol	1907	Alcohols	4.43	<0.001
22	Isovaleral	912	Aldehydes	3.54	<0.001
23	Pentanal	972	Aldehydes	5.44	<0.001
24	Hexanal	1076	Aldehydes	3.33	<0.001
26	Heptanal	1180	Aldehydes	2.60	<0.001
32	2,4-Heptadien-1-al	1486	Aldehydes	2.85	<0.001
51	2-Isopropyltoluene	1263	Aromatic hydrocarbons	2.46	<0.001
65	Bornyl acetate	1577	Esters	2.31	<0.001
71	Triisobutyl phosphate	1918	Esters	3.09	0.03
76	2-Ethylfuran	945	Furans	2.04	<0.001
77	2-Amylfuran	1228	Furans	2.25	<0.001
79	2-Butanone	900	Ketones	2.05	<0.001
80	Isopropylideneacetone	1126	Ketones	3.94	<0.001
84	Hydroxyacetone	1290	Ketones	2.74	<0.001
85	2,6,6-Trimethylcyclohexanone	1302	Ketones	2.30	<0.001
88	Diacetone alcohol	1356	Ketones	2.09	<0.001
100	Cyclofenchene	1016	Monoterpenes	5.84	<0.001
101	Limonene	1191	Monoterpenes	3.95	<0.001
103	Linalool	1548	Monoterpenes	4.21	<0.001
108	*cis*-Linalool 3,7-oxide	1763	Monoterpenes	2.65	<0.001
109	Geraniol	1848	Monoterpenes	4.21	<0.001
112	2-Ethylpyrrole	1174	Nitrogen heterocycles	2.59	<0.001
123	Butylated Hydroxytoluene	1909	Phenols	3.11	<0.001
134	Dimethyl Sulfoxide	1570	Others	3.73	<0.001

a Compound numbers correspond to those listed in Table S1.

b Linear retention indices (LRIs) were calculated on a DB-Wax column using a series of C_7_-C_30_*n*-alkanes as reference standards.

c Variable importance in projection (VIP) values were derived from the PLS-DA model based on the HS-SPME-GC–MS dataset.

d *p* value was calculated by one-way analysis of variance (ANOVA).

**Table 2:** Preliminary grade-associated non-volatile candidate features identified by exploratory screening.

No.^a^	Compound	RT^b^	Class	VIP value^c^	*p* value^d^
Positive mode					
9	Theophylline	208.9	Alkaloids	5.81	<0.001
12	Caffeine	242.5	Alkaloids	9.77	<0.001
18	Linoleamide	526.9	Amides	6.02	<0.001
42	Theanine	52.8	Amino acids and peptides	7.53	<0.001
47	Leucine	63.2	Amino acids and peptides	3.89	<0.001
50	Phenylalanine	100.7	Amino acids and peptides	3.11	<0.001
53	Tryptophan	206.3	Amino acids and peptides	2.76	<0.001
155	(+)-Gallocatechin	212.8	Flavonoids	2.00	<0.001
157	Epigallocatechin	235.4	Flavonoids	4.31	<0.001
159	Procyanidin B2	241.3	Flavonoids	3.26	<0.001
164	Epigallocatechin gallate/Gallocatechin gallate	258.7	Flavonoids	2.70	<0.001
165	Corymboside/Isoschaftoside/Schaftoside/Vicenin 3	267.2	Flavonoids	2.68	<0.001
171	(−)-Catechin gallate	282.7	Flavonoids	4.11	<0.001
176	Rutin	292.3	Flavonoids	2.48	<0.001
185	Nicotiflorin	306.7	Flavonoids	3.68	<0.001
204	LPC(18:2/0:0)	495.9	Glycerophospholipids	2.94	<0.001
208	LPC(16:0)	513.9	Glycerophospholipids	2.37	<0.001
218	PC(36:6)	590.4	Glycerophospholipids	3.67	<0.001
223	PC(36:5)	607.3	Glycerophospholipids	3.84	<0.001
253	Adenosine	186.4	Nucleotides and derivatives	3.59	<0.001
308	1-Deoxypebrolide	429.2	Terpenoids	2.89	<0.001
Negative mode					
39	Pyroglutamic acid	46.6	Amino acids and peptides	3.53	<0.001
42	Theanine	52.8	Amino acids and peptides	4.91	<0.001
47	Leucine	63.2	Amino acids and peptides	2.67	<0.001
53	Tryptophan	206.3	Amino acids and peptides	2.45	<0.001
54	*N*-Acetyltryptophan	274.6	Amino acids and peptides	2.76	<0.001
58	Corilagin	235.0	Aromatic acids	4.72	<0.001
60	Methylgallate	246.0	Aromatic acids	4.24	<0.001
75	Isomaltose/Gentiobiose/Trehalose/Palatinose/Galactinol/Neotrehalose/Turanose/Sucrose	44.2	Carbohydrates	2.18	<0.001
135	12-Hydroxystearic acid	511.8	Fatty acids and derivatives	2.71	<0.001
139	*α*-Linolenic acid	534.3	Fatty acids and derivatives	2.54	<0.001
155	(+)-Gallocatechin	212.8	Flavonoids	2.72	<0.001
157	Epigallocatechin	235.4	Flavonoids	5.32	<0.001
158	Leucocyanidin	235.6	Flavonoids	5.32	<0.001
159	Procyanidin B2	241.3	Flavonoids	5.75	<0.001
175	Quercetin-3′-*β*-d-glucopyranosyl-(1 → 2)-*α*-L-rhamnopyranoside	292.3	Flavonoids	2.99	<0.001
176	Rutin	292.3	Flavonoids	2.99	<0.001
182	Kaempferol-3-*O*-galactoside	304.0	Flavonoids	4.64	<0.001
184	Kaempferol 3-*O*-*β*-d-glucopyranosyl-(1 → 2)-*α*-L-rhamnopyranoside	306.3	Flavonoids	3.67	<0.001
192	Quercetin	330.1	Flavonoids	2.12	<0.001
194	Tiliroside	342.4	Flavonoids	2.09	<0.001
258	Malic acid	46.1	Organic acids and derivatives	2.73	<0.001
260	Isocitric acid	46.7	Organic acids and derivatives	4.68	<0.001
261	Citric Acid	57.9	Organic acids and derivatives	2.59	<0.001
275	5-*O*-Coumaroylquinic acid	230.1	Phenylpropanoids	3.92	<0.001
276	Chlorogenic acid	235.4	Phenylpropanoids	2.92	<0.001
278	Scopolin	236.8	Phenylpropanoids	2.92	<0.001
279	4-*p*-Coumaroylquinic acid	253.4	Phenylpropanoids	6.64	<0.001
287	3-(2,4-Dihydroxypentyl)-6,8-dihydroxyisochromen-1-one	288.5	Phenylpropanoids	5.74	<0.001
303	Acuminoside/Kenposide A/Geranyl *β*-primeveroside	362.3	Terpenoids	2.30	<0.001

a Feature numbers correspond to those listed in Table S2.

b RT: retention time (s).

c Variable importance in projection (VIP) values were obtained from the PLS-DA model based on the UPLC-QE-Orbitrap-HRMS dataset.

d *p* values were calculated using one-way analysis of variance (ANOVA).

To further identify candidate features showing grade-related abundance trends, their relative abundance patterns across the ordered G1-G6 sequence were examined using heatmaps (Fig. 4). Several candidate features showed overall abundance trends across the grade sequence. Among the volatile compounds, hexanal (24) and heptanal (26) generally decreased toward the lower commercial grades, whereas 2,6,6-trimethylcyclohexanone (85) generally increased across the grade sequence (Fig. 4A). In the LC–MS datasets, epigallocatechin (157) generally increased toward the lower grades in both positive and negative ion modes, and (+)-gallocatechin (155) showed a similar overall trend (Fig. 4B and C). In contrast, theanine (42) showed an overall decreasing tendency toward the lower grades, particularly in the negative ion mode. Previous studies have shown that the metabolite composition of green tea varies with plucking position and plucking period, with younger leaves generally containing higher levels of theanine and lower levels of several catechins, whereas delayed plucking is accompanied by decreased theanine and increased epigallocatechin (Lee et al., 2011; Lee, Kim, Kim, & Kim, 2014). The decreasing tendency of theanine and the overall increases in (+)-gallocatechin and epigallocatechin observed toward the lower grades are therefore broadly consistent with previously reported maturity-related changes and may partly reflect differences in raw-leaf maturity associated with grade-specific plucking standards. Previous studies have also shown that theanine contributes to the umami taste of tea, whereas catechins are important contributors to bitterness and astringency (Xu et al., 2018; Zhang, Cao, Granato, & Xu, 2020). Thus, the grade-related variation in theanine and catechins observed here may also be relevant to differences in taste characteristics among the six GT grades. Similarly, hexanal and heptanal are aroma-related volatile compounds (Table S1), and their grade-related abundance changes may be associated with differences in aroma characteristics among GT grades. However, raw-leaf maturity and sensory attributes were not directly measured in the present study, and these proposed relationships remain tentative and require further investigation.

**Fig. 4:**
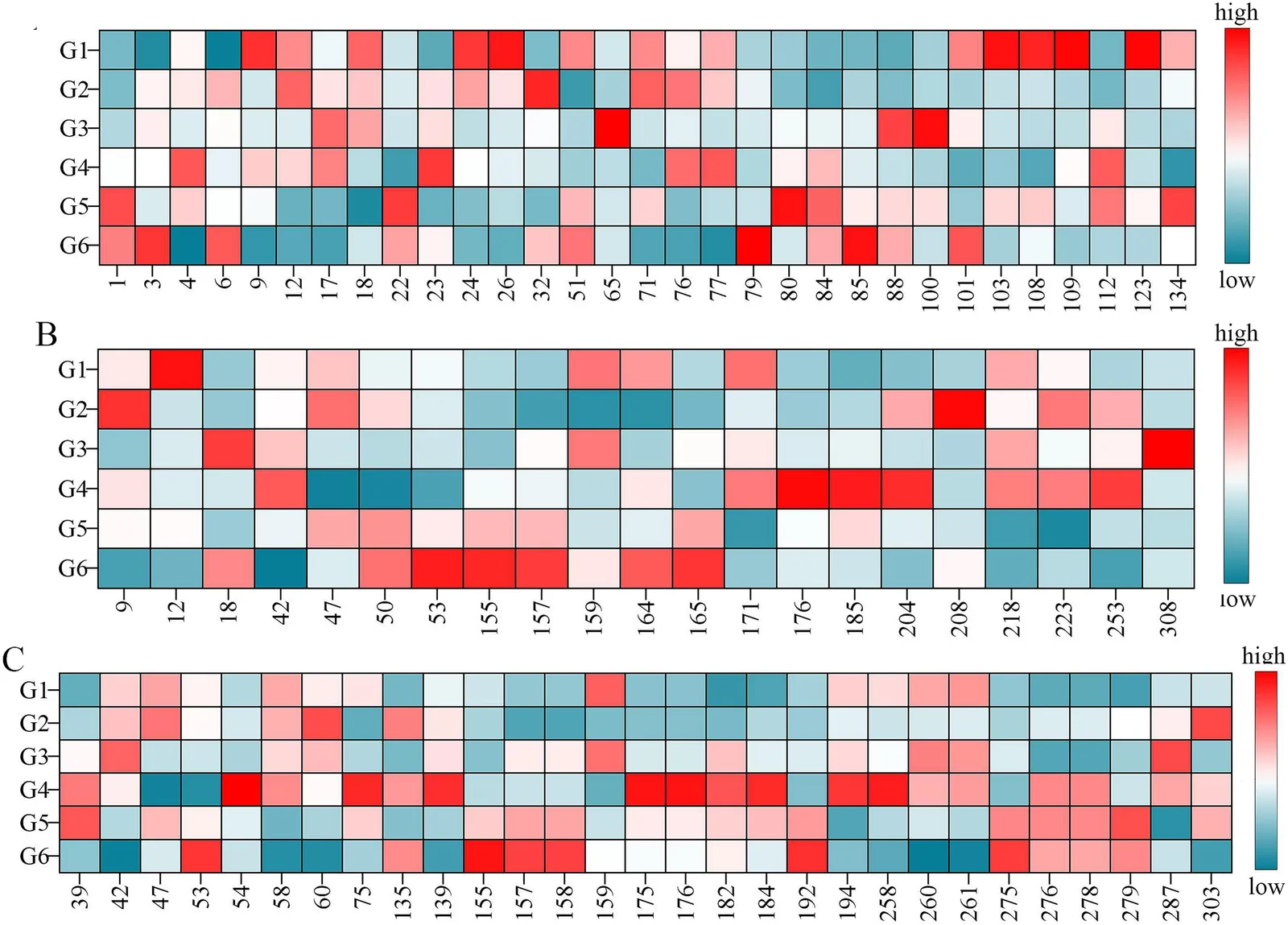
Heatmaps showing the relative abundance patterns of grade-associated candidate features detected in GT samples across six grades. (A) GC–MS dataset; (B) LC–MS positive ion mode dataset; (C) LC–MS negative ion mode dataset. Feature numbers correspond to those listed in Table S1 for panel A and Table S2 for panels B and C.

By contrast, some candidate features exhibited non-linear abundance patterns across the six grades. For example, *α*-linolenic acid (139) followed the abundance order G4 > G3 > G2 > G1 > G5 > G6, rather than showing a consistent directional change along the G1–G6 sequence. Overall, the candidate metabolites identified in the present study provide preliminary evidence of grade-associated chemical variation within the investigated samples. However, all samples were collected from a single plantation and a single harvest year, and samples from different production batches within each grade were pooled before instrumental analysis. The general applicability of these features across production batches, plantations, and harvest years therefore remains to be established. In addition, no independent validation set was included in the present study. Accordingly, these features should be considered candidate grade-associated metabolites rather than validated authentication markers. Further studies based on independent, unpooled production batches, appropriate multiple-testing correction, and external validation are needed to assess their reproducibility and value for grade authentication.

### 3.5 Antioxidant evaluations of GT samples

Tea is widely recognized for its antioxidant properties, although antioxidant capacity can vary with tea type, cultivar, processing grade, and extraction conditions (Anesini, Ferraro, & Filip, 2008; Cabrera et al., 2006). In this study, the antioxidant activities of GT samples across six commercial grades were evaluated using DPPH, ABTS, and FRAP assays. All GT samples showed strong radical-scavenging activities. In the DPPH assay, the inhibition rates of all samples exceeded 84% at a final concentration of 50 μg/mL, with no significant differences among grades (*p* > 0.05) (Fig. S1). Further IC_50_ determination revealed that the DPPH IC_50_ values ranged from 4.50 to 5.78 μg/mL (Fig. 5A). Among the six grades, G6 exhibited the strongest DPPH radical-scavenging activity, with the lowest IC_50_ value of 4.50 μg/mL, followed by G4 (4.74 μg/mL) and G3 (5.02 μg/mL). These values were lower than those of the positive control LAA (IC_50_ = 7.88 μg/mL) under the same assay conditions, indicating the strong DPPH radical-scavenging capacity of GT extracts (Fig. 5A). A similar pattern was observed in the ABTS assay. At a final concentration of 50 μg/mL, the ABTS radical-scavenging rates ranged from 74% to 82%, with no significant differences among grades (*p* > 0.05) (Fig. S1). The ABTS IC_50_ values varied from 6.79 to 9.30 μg/mL, with G6 showing the strongest activity (IC_50_ = 6.79 μg/mL), followed by G4 (7.39 μg/mL) and G3 (7.77 μg/mL) (Fig. 5B). These IC_50_ values were also lower than that of LAA (13.63 μg/mL), further supporting the pronounced radical-scavenging potential of GT samples. The reducing capacities of GT samples were further assessed using the FRAP assay (Fig. 5C). All samples exhibited concentration-dependent ferric-reducing activities. Among the tested grades, G1, G4, and G6 generally showed stronger reducing capacities, with FRAP values ranging from 78.38 to 84.20 μmol Fe^2+^/L at the tested concentration of 12.50 μg/mL (Fig. 5C). These results, together with the DPPH and ABTS data, indicate that GT possesses considerable antioxidant potential. Compared with previously reported values for various green tea extracts, which showed DPPH IC_50_ values of 16.30–226.28 μg/mL and ABTS IC_50_ values of 14.17–117.62 μg/mL (Deo et al., 2024; Korkmaz et al., 2019; Kumari & Kumar, 2022), the lower IC_50_ values observed in the present study suggest the excellent antioxidant capacity of GT. Notably, the antioxidant activities of GT samples were not consistent with the descending order of commercial grade, suggesting that antioxidant capacity is not directly reflected by commercial quality level. This discrepancy may be related to the fact that commercial grade is mainly associated with plucking standard, leaf tenderness, and sensory quality, as defined by GB/T 19691–2008, whereas antioxidant capacity is more closely related to the accumulation of specific antioxidant-related metabolites. The strong antioxidant activities observed in GT may be partly associated with the presence of multiple antioxidant-related metabolites annotated by LC–MS, including quercetin (Boots, Haenen, & Bast, 2008), epigallocatechin gallate (Zhong & Shahidi, 2012), ellagic acid (Priyadarsini, Khopde, Kumar, & Mohan, 2002), and chlorogenic acid (Sato et al., 2011), which have been reported to possess significant antioxidant properties. These compounds, together with other phenolic constituents, may partly contribute to the radical-scavenging and ferric-reducing capacities of GT samples.

**Fig. 5:**
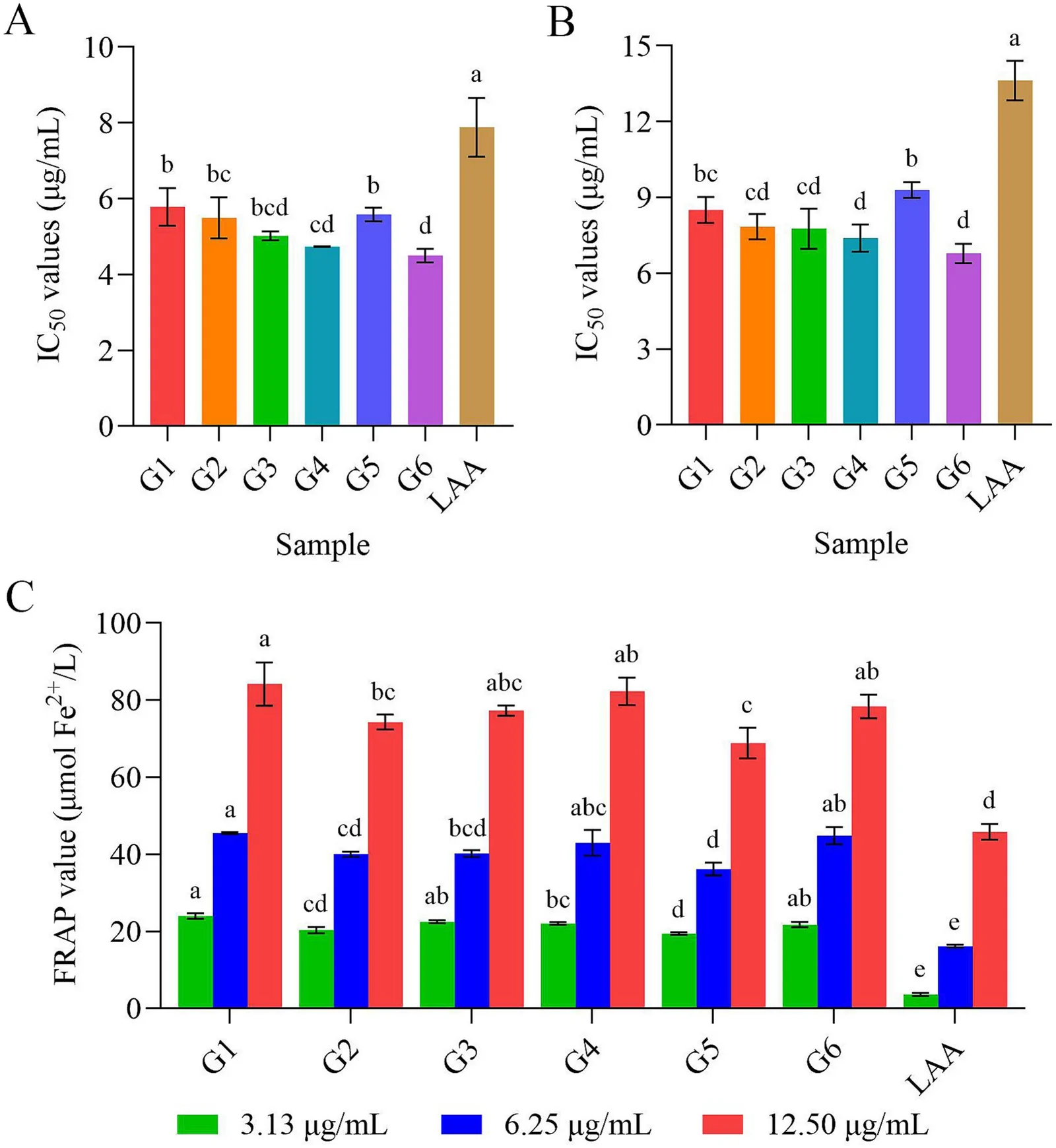
Antioxidant activities of different grades of GT samples. (A) DPPH radical scavenging activity expressed as IC_50_ values. (B) ABTS radical scavenging activity expressed as IC_50_ values. (C) FRAP values of GT samples at different concentrations. Data are presented as mean ± SD (*n* = 3). LAA, l-ascorbic acid. Different lowercase letters indicate significant differences among GT samples within the same assay (A and B) or within the same concentration level (C), as determined by one-way ANOVA followed by Duncan's multiple comparison test (*p* < 0.05).

### 3.6 Associations between metabolites and antioxidant potential

To further explore the relationships between metabolite variation and antioxidant activity in GT, Spearman correlation analysis was performed between grade-associated metabolites annotated by UPLC-QE-Orbitrap-HRMS and antioxidant indices derived from the DPPH, ABTS, and FRAP assays across the six GT grades (*n* = 6) (Fig. 6 and Table S4). Both positive and negative correlations were observed in the positive and negative ion mode datasets, indicating complex relationships between metabolite variation and antioxidant activity (Fig. 6). Several metabolites showed positive associations with antioxidant capacity across the three assays, including PC(36:6) (218) and 1-deoxypebrolide (308) in positive ion mode (Fig. 6A) and (+)-gallocatechin (155) in negative ion mode (Fig. 6B). Among them, (+)-gallocatechin has previously been reported to possess free radical-scavenging activity (Nakagawa & Yokozawa, 2002). However, the antioxidant relevance of some metabolites, such as PC(36:6) and 1-deoxypebrolide, remains unclear and requires further exploration. In addition, several flavonoids were positively associated with DPPH, ABTS or FRAP assays, including (−)-catechin gallate (171) and rutin (176) in positive ion mode, as well as epigallocatechin (157) and kaempferol-3-O-galactoside (182) in negative ion mode. Antioxidant activities of rutin, (−)-catechin gallate, and epigallocatechin have been reported previously (Ambigaipalan, Oh, & Shahidi, 2020; Guo et al., 1999; Yang, Guo, & Yuan, 2008), implying that they might be candidate metabolites associated with antioxidant activity in GT. In contrast, several amino acids, terpenoids, and alkaloids showed negative associations with antioxidant capacity. For example, caffeine (12) was negatively associated with DPPH and ABTS antioxidant indices (Fig. 6A), although it has been reported to exhibit antioxidant properties in previous studies (Brezová, Šlebodová, & Staško, 2009). When statistical significance was considered, only the negative correlations of caffeine with DPPH (*p* = 0.019) and ABTS (*p* = 0.042) reached *p* < 0.05, whereas the other correlations were not statistically significant (Table S4). The limited number of significant correlations may partly reflect the small number of grade-level observations (*n* = 6) and the resulting limited statistical power. Therefore, these associations should be interpreted as exploratory and hypothesis-generating rather than as evidence of direct contributions of individual metabolites to the antioxidant activity of GT. In addition, the LC–MS data were based on relative peak areas rather than absolute quantification. Differences in ionization efficiency and matrix effects can result in compound-dependent MS responses, limiting direct comparison of peak areas among different metabolites. Targeted quantification and validation in larger independent sample sets are therefore needed to confirm the observed associations.

**Fig. 6:**
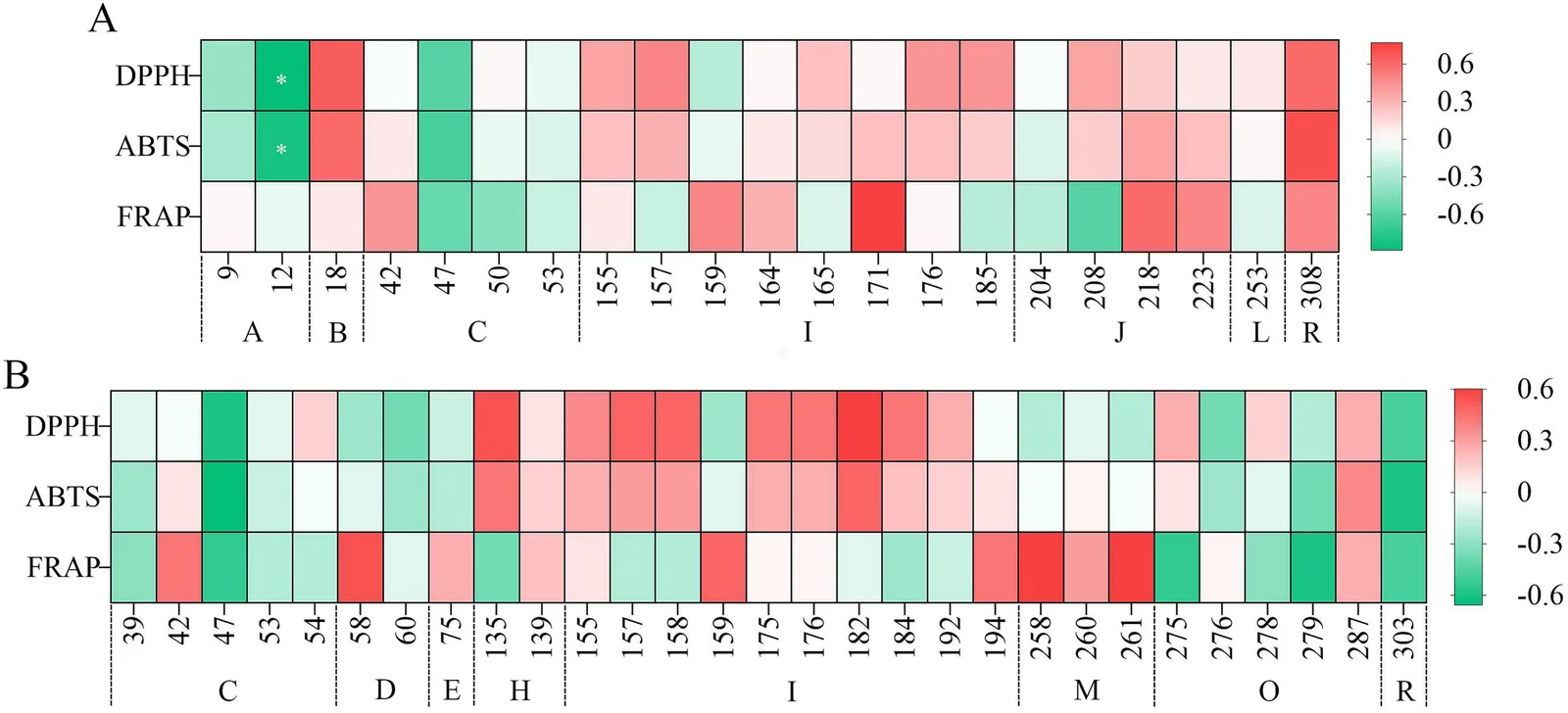
Spearman correlation analysis between grade-associated non-volatiles identified by UPLC-QE-Orbitrap-HRMS and antioxidant activities. (A) Positive ion mode. (B) Negative ion mode. Color intensity represents the Spearman correlation coefficient, with red indicating positive correlations and green indicating negative correlations. Correlation analysis was based on six grade-level observations (*n* = 6) and was considered exploratory and hypothesis-generating. Asterisks indicate nominally significant two-tailed correlations (*p* < 0.05). Dashed lines separate different metabolite classes, with the letters corresponding to the class codes used in Table S2: A, alkaloids; B, amides; C, amino acids and peptides; D, aromatic acids; E, carbohydrates; H, fatty acids and derivatives; I, flavonoids; J, glycerophospholipids; L, nucleotides and derivatives; M, organic acids and derivatives; O, phenylpropanoids; and R, terpenoids. (For interpretation of the references to color in this figure legend, the reader is referred to the web version of this article.)

## 4 Conclusions

This study systematically investigated the grade-dependent chemical variation and antioxidant activities of GT using an integrated HS-SPME-GC–MS and UPLC-QE-Orbitrap-HRMS metabolomics strategy combined with chemometric analysis. A total of 137 volatile compounds and 328 non-volatile features were characterized across six commercial grades of GT, demonstrating the considerable chemical diversity of this regional green tea. Alcohols, aldehydes, and ketones were the dominant volatile classes, whereas fatty acid derivatives, flavonoids, and amino acids and peptides represented the frequently detected non-volatile constituents. Multivariate analyses revealed grade-associated metabolic differences, indicating that volatile and non-volatile profiles provide complementary chemical information for grade differentiation. A total of 81 features, including 31 volatile and 50 non-volatile features, were preliminarily screened as candidate grade-associated features. Among them, 1-penten-3-ol, caffeine, theanine, epigallocatechin, procyanidin B2, and several catechin-related compounds were among the prominent candidate features associated with commercial grade variation. All GT samples exhibited strong antioxidant activities, with lower DPPH and ABTS IC_50_ values and higher FRAP values than LAA under the same assay conditions, indicating considerable antioxidant potential. Among them, G6 exhibited the lowest IC_50_ values, whereas G1 exhibited the highest ferric-reducing capacity in the FRAP assay. Exploratory correlation analysis further showed positive associations between several flavonoids, including (−)-catechin gallate, rutin, and epigallocatechin, and antioxidant capacity, suggesting their potential as candidate metabolites associated with antioxidant activity. Overall, this work provides comprehensive metabolomic evidence for the chemical differentiation of GT across commercial grades. Future studies involving targeted quantification, independent sample validation, and experimental verification of candidate metabolites would further clarify their relationships with GT quality and antioxidant activity.

## CRediT authorship contribution statement

**Fengke Lin:** Writing – original draft, Methodology, Funding acquisition, Formal analysis, Data curation, Conceptualization. **Qun Zhou:** Data curation. **Nuo Chen:** Data curation. **Ying Ren:** Data curation. **Wenkai Zhao:** Data curation. **Jianbo Nie:** Data curation. **Yixin Qin:** Data curation. **Trevor N. Clark:** Writing – review & editing, Validation. **Chunsong Cheng:** Writing – review & editing, Validation, Funding acquisition. **Binsheng Luo:** Writing – review & editing, Supervision, Project administration, Funding acquisition, Formal analysis, Conceptualization.

## Declaration of competing interest

The authors declare that they have no known competing financial interests or personal relationships that could have appeared to influence the work reported in this paper.

## Data Availability

The raw GC–MS and LC–MS datasets generated in this study have been deposited in the MetaboLights repository under accession numbers MTBLS16075 and MTBLS16089, respectively. Additional data supporting the findings of this study are provided in the article and its Supplementary Materials.

## Appendix A Supplementary data

Supplementary material Supplementary data include Table S1, volatile compounds characterized in GT samples across different commercial grades; Table S2, non-volatile metabolites characterized by UPLC-QE-Orbitrap-HRMS-based metabolomics; Table S3, quality parameters of the PLS-DA models; Table S4, Spearman correlations between grade-associated non-volatile features and antioxidant indices; and Fig. S1, DPPH and ABTS radical-scavenging activities of GT samples.
