# Supplementary material for: Integrated GC–MS and LC–MS metabolomics reveals grade-dependent chemical variation and antioxidant activity of Gougunao tea
Source: Food Chem X. 2026 Oct 29;40:104721. doi: 10.1016/j.fochx.2026.104721 (PMC13742212; doi:10.1016/j.fochx.2026.104721)
Supplement: Supplementary file 1 [file mmc1.docx]

**Table S1.** Volatile compounds characterized in GT samples across different commercial grades.

| No. | Compound*^a^* | Class | LRI*^b^* | LRI*^c^* | Relative abundance (%)*^d^* | | | | | | Odor |
| --- | --- | --- | --- | --- | --- | --- | --- | --- | --- | --- | --- |
|  |  |  |  |  | G1 | G2 | G3 | G4 | G5 | G6 |  |
| 1 | 1-Methoxypropan-2-ol | Alcohols | 1125 | 1135 | 0.38 | 0.39 | 0.49 | 0.63 | 1.02 | 0.91 | Pleasant, Mild, Ethereal |
| 2 | Butanol | Alcohols | 1146 | 1150 | 0.18 | 0.25 | 0.35 | 0.28 | 0.22 | 0.30 | Medicine, Fruity |
| 3 | 1-Penten-3-ol | Alcohols | 1161 | 1161 | 8.38 | 14.46 | 14.62 | 14.12 | 13.13 | 20.10 | Butter, Pungent, Green |
| 4 | Pentanol | Alcohols | 1252 | 1255 | 2.75 | 2.79 | 2.63 | 3.23 | 2.87 | 2.07 | Fruity, Balsamic, Vanilla, Fusel |
| 5 | Ethriol | Alcohols | 1312 |  | 0.02 | 0.02 | 0.02 | 0.02 | 0.03 | 0.03 |  |
| 6 | *cis*-2-Pentenol | Alcohols | 1320 | 1322 | 1.68 | 2.86 | 2.57 | 2.47 | 2.55 | 3.24 | Rubber, Green, Fruity |
| 7 | 1,2-Butanediol | Alcohols | 1334 |  | 0.33 | 0.49 | 0.41 | 0.37 | 0.31 | 0.43 |  |
| 8 | Hexanol | Alcohols | 1354 | 1356 | 0.74 | 0.45 | 0.49 | 0.44 | 0.55 | 0.29 | Resin, Flower, Green |
| 9 | *cis*-3-Hexene-1-ol | Alcohols | 1384 | 1389 | 2.89 | 1.60 | 1.64 | 2.07 | 1.76 | 0.87 | Leafy, Grass, Vegetable, Herbal |
| 10 | Butyl oxitol | Alcohols | 1400 | 1405 | tr | 0.01 | 0.01 | 0.04 | 0.03 | tr | Rancid, Weak, Pleasant |
| 11 | 2-Hexenol | Alcohols | 1406 | 1410 | 0.07 | 0.05 | 0.06 | 0.01 | 0.02 | 0.01 | Leafy, Green, Wine, Fruity |
| 12 | Morillol | Alcohols | 1452 | 1456 | 1.20 | 1.30 | 0.82 | 1.01 | 0.56 | 0.52 | Raw, Fishy, Oily, Earthy |
| 13 | 1-Octanol | Alcohols | 1560 | 1564 | 0.12 | 0.10 | 0.10 | 0.10 | 0.11 | 0.07 | Burnt, Orange, Rose, Waxy |
| 14 | *trans*-2-Octenol | Alcohols | 1616 | 1620 | 0.08 | 0.09 | 0.07 | 0.08 | 0.02 | 0.03 |  |
| 15 | 1,2-Ethanediol | Alcohols | 1623 | 1621 | 0.09 | 0.05 | 0.04 | 0.06 | 0.15 | 0.05 | Odorless |
| 16 | Furfuralcohol | Alcohols | 1657 | 1656 | 0.09 | 0.06 | 0.10 | 0.11 | 0.18 | 0.06 | Coffee, Alcoholic, Bitter, Musty |
| 17 | Phenylmethanol | Alcohols | 1872 | 1877 | 1.77 | 1.95 | 2.47 | 2.37 | 1.31 | 1.15 | Berry, Balsamic, Floral, Sweet, |
| 18 | Benzeneethanol | Alcohols | 1907 | 1912 | 4.64 | 4.10 | 4.29 | 3.47 | 2.74 | 3.57 | Lilac, Rose, Honey, Floral, Spice |
| 19 | Arosol | Alcohols | 2137 | 2142 | 0.03 | 0.03 | 0.06 | 0.04 | 0.06 | 0.03 | Faint aromatic odor |
| 20 | Butanal | Aldehydes | 885 | 890 | 0.24 | 0.34 | 0.33 | 0.34 | 0.22 | 0.46 | Bready, Chocolate, Creamy, Pear |
| 21 | *α*-Methylbutanal | Aldehydes | 909 | 908 | 0.03 | 0.03 | 0.02 | 0.03 | 0.06 | 0.05 | Musty, Coffee, Cocoa, Nutty |
| 22 | Isovaleral | Aldehydes | 912 | 912 | 1.68 | 1.72 | 1.68 | 1.16 | 2.68 | 2.25 | Peach, Sour, Fatty |
| 23 | Pentanal | Aldehydes | 972 | 974 | 11.70 | 13.55 | 13.57 | 15.33 | 11.85 | 13.35 | Almond, Malt, Pungent |
| 24 | Hexanal | Aldehydes | 1076 | 1079 | 2.41 | 1.82 | 0.98 | 1.28 | 0.68 | 0.61 | Leafy, Sweaty, Tallow, Fresh |
| 25 | *trans*-2-Pentenal | Aldehydes | 1121 | 1125 | 0.01 | 0.02 | 0.01 | 0.01 | tr | nd | Apple, Orange, Strawberry |
| 26 | Heptanal | Aldehydes | 1180 | 1181 | 2.10 | 1.18 | 0.89 | 0.95 | 0.78 | 0.44 | Citrus, Herbal |
| 27 | 2-Hexenal | Aldehydes | 1210 | 1216 | 0.19 | 0.34 | 0.21 | 0.20 | 0.12 | 0.34 | Apple, Green |
| 28 | *cis*-4-Heptenal | Aldehydes | 1236 | 1236 | 0.10 | 0.28 | 0.19 | 0.18 | 0.11 | 0.24 | Cream, Biscuit, Milky |
| 29 | Octanal | Aldehydes | 1284 | 1287 | 0.09 | 0.07 | 0.04 | 0.05 | 0.04 | 0.03 | Lemon, Citrus, Orange |
| 30 | 3-Butylacrolein | Aldehydes | 1316 | 1318 | 0.02 | 0.03 | 0.10 | 0.17 | 0.16 | 0.04 | Soap, Vegetable, Fresh, Fatty |
| 31 | *trans*-2-Octenal | Aldehydes | 1423 | 1425 | 0.01 | 0.02 | 0.01 | 0.01 | 0.01 | 0.01 | Nut, Fatty, Herbal, Fresh, Green |
| 32 | 2,4-Heptadien-1-al | Aldehydes | 1486 | 1487 | 0.91 | 1.55 | 1.12 | 1.06 | 0.90 | 1.24 | Hazelnut, Cinnamon, Aldehydic |
| 33 | (2*E*,6*Z*)-Nonadienal | Aldehydes | 1580 | 1587 | 0.03 | 0.05 | 0.04 | 0.03 | 0.02 | 0.07 | Wax, Fatty, Cucumber, Leafy |
| 34 | Phenylethanal | Aldehydes | 1633 | 1636 | 0.05 | 0.03 | 0.03 | 0.02 | 0.08 | 0.10 | Hyacinth, Honey, Clover, Sweet |
| 35 | 4-Oxohex-2-enal | Aldehydes | 1748 |  | 0.16 | 0.25 | 0.15 | 0.14 | 0.09 | 0.21 |  |
| 36 | 3,4-Dimethylbenzaldehyde | Aldehydes | 1801 |  | 0.17 | 0.18 | 0.15 | 0.18 | 0.12 | 0.13 |  |
| 37 | 3-Methylnonane | Alkanes | 962 | 983 | 0.02 | 0.02 | 0.03 | 0.03 | 0.07 | 0.06 |  |
| 38 | 2,6-Dimethyloctane | Alkanes | 1010 |  | 1.06 | 1.39 | 1.26 | 1.34 | 0.94 | 0.98 |  |
| 39 | 3-Ethyl-3-methylheptane | Alkanes | 1034 |  | 0.01 | 0.01 | 0.01 | nd | tr | tr |  |
| 40 | 4-Ethyloctane | Alkanes | 1048 |  | tr | tr | 0.01 | 0.02 | 0.01 | 0.01 |  |
| 41 | 5-Methyldecane | Alkanes | 1049 |  | 0.01 | 0.01 | 0.01 | 0.01 | 0.01 | 0.01 |  |
| 42 | 2-Methyldecane | Alkanes | 1055 | 1053 | 0.11 | 0.12 | 0.15 | 0.14 | 0.10 | 0.08 |  |
| 43 | 2-Ethylnonane | Alkanes | 1062 | 1048 | 0.10 | 0.19 | 0.20 | 0.12 | 0.15 | 0.14 |  |
| 44 | 2-Methyltridecane | Alkanes | 1357 |  | 0.05 | 0.05 | 0.05 | 0.05 | 0.03 | 0.06 |  |
| 45 | 2,4-Dimethyl-1-heptene | Alkenes | 890 |  | 0.02 | 0.07 | 0.02 | 0.03 | 0.30 | 0.03 |  |
| 46 | 2,6-Dimethylstyrene | Alkenes | 1466 |  | 0.14 | 0.15 | 0.14 | 0.16 | 0.08 | 0.11 |  |
| 47 | Ethylacetamide | Amides | 1630 | 1608 | 0.03 | 0.02 | 0.04 | 0.05 | 0.05 | 0.03 |  |
| 48 | Ethylformamide | Amides | 1647 |  | 0.07 | 0.06 | 0.09 | 0.12 | 0.07 | 0.04 |  |
| 49 | Formanilide | Amides | 2156 |  | tr | tr | 0.01 | 0.01 | 0.01 | tr |  |
| 50 | Ethylbenzene | Aromatic hydrocarbons | 1175 | 1168 | 0.14 | 0.24 | 0.26 | 0.09 | 0.08 | 0.21 | Aromatic, Pungent, Sweet |
| 51 | 2-Isopropyltoluene | Aromatic hydrocarbons | 1263 | 1268 | 0.70 | 0.27 | 0.42 | 0.40 | 0.63 | 0.73 |  |
| 52 | Mesitylene | Aromatic hydrocarbons | 1272 | 1283 | 0.19 | 0.13 | 0.14 | 0.15 | 0.15 | 0.12 | Peculiar |
| 53 | 2-Methyl-*p*-ethyltoluene | Aromatic hydrocarbons | 1352 | 1354 | 0.29 | 0.31 | 0.29 | 0.33 | 0.22 | 0.23 |  |
| 54 | Durene | Aromatic hydrocarbons | 1427 | 1438 | 0.92 | 1.00 | 0.92 | 1.03 | 0.68 | 0.73 | Rancid, Sweet |
| 55 | Pentamethylbenzene | Aromatic hydrocarbons | 1490 |  | 0.17 | 0.18 | 0.16 | 0.16 | 0.11 | 0.13 |  |
| 56 | Naphthalene | Aromatic hydrocarbons | 1729 | 1732 | 0.32 | 0.38 | 0.35 | 0.42 | 0.28 | 0.32 | Pungent, Tarry, Aromatic |
| 57 | 2-Methylnaphthalene | Aromatic hydrocarbons | 1841 | 1852 | 0.07 | 0.09 | 0.07 | 0.08 | 0.05 | 0.07 |  |
| 58 | Acenaphthylene | Aromatic hydrocarbons | 2180 |  | tr | nd | tr | tr | 0.01 | tr |  |
| 59 | Ethyl acetate | Esters | 892 | 887 | 0.03 | 0.04 | 0.06 | 0.04 | 0.06 | 0.06 | Pineapple, Sweet, Anise, Fruity |
| 60 | Butyl propionate | Esters | 1138 | 1138 | 0.04 | 0.04 | 0.07 | 0.08 | 0.03 | 0.03 | Rose, Banana, Earthy |
| 61 | Methyl caproate | Esters | 1183 | 1181 | 0.03 | 0.03 | 0.03 | 0.04 | 0.04 | 0.02 | Pineapple, Fresh, Fruity, Sweet |
| 62 | Butyl butanoate | Esters | 1216 | 1212 | 0.19 | 0.17 | 0.22 | 0.34 | 0.13 | 0.13 | Peach, Apple, Berry, Pineapple |
| 63 | *trans*-3-Hexenyl butyrate | Esters | 1459 | 1452 | 0.18 | nd | nd | nd | 0.06 | nd |  |
| 64 | Ethyl 3-hydroxybutyrate | Esters | 1515 | 1522 | nd | nd | nd | nd | 0.02 | nd | Marshmallow, Apple Skin, Grape |
| 65 | Bornyl acetate | Esters | 1577 | 1570 | 0.02 | tr | 0.17 | 0.01 | 0.02 | 0.02 |  |
| 66 | Hexanoic anhydride | Esters | 1654 |  | 0.13 | 0.12 | 0.08 | 0.08 | 0.02 | 0.03 |  |
| 67 | *cis*-3-Hexenyl hexanoate | Esters | 1656 | 1648 | 0.50 | 0.06 | 0.06 | 0.02 | 0.21 | 0.01 | Pineapple, Prune, Waxy, Winey |
| 68 | Ethyl benzoate | Esters | 1660 | 1652 | 0.02 | 0.03 | 0.04 | 0.02 | 0.02 | 0.04 | Celery, Wintergreen, Grape |
| 69 | Benzylcarbinyl formate | Esters | 1805 | 1784 | 0.03 | tr | 0.01 | 0.01 | 0.01 | 0.01 | Hyacinth, Rose, Green, Herbal |
| 70 | 3-Hydroxy-2,2,4-trimethylpentyl isobutyrate | Esters | 1866 |  | 0.01 | 0.02 | 0.02 | 0.02 | 0.02 | 0.02 |  |
| 71 | Triisobutyl phosphate | Esters | 1918 |  | 1.76 | 1.87 | 1.33 | 1.14 | 1.57 | 1.05 |  |
| 72 | Phenethyl isovalerate | Esters | 1959 | 1980 | 0.01 | nd | nd | nd | nd | nd | Rose, Peach, Apricot, Sweet |
| 73 | Butyl ether | Ethers | 960 | 974 | 0.05 | 0.07 | 0.09 | 0.10 | 0.06 | 0.04 | Ethereal |
| 74 | Anethole | Ethers | 1820 | 1820 | 0.05 | 0.05 | 0.05 | 0.03 | 0.04 | 0.11 |  |
| 75 | 2-Methylfuran | Furans | 883 | 898 | 0.05 | 0.06 | 0.06 | 0.07 | 0.05 | 0.05 | Ethereal, Acetone, Chocolate |
| 76 | 2-Ethylfuran | Furans | 945 | 945 | 0.64 | 0.80 | 0.59 | 0.81 | 0.48 | 0.42 | Earthy, Sweet, Burnt, Malty |
| 77 | 2-Amylfuran | Furans | 1228 | 1231 | 0.85 | 0.81 | 0.65 | 0.98 | 0.64 | 0.44 | Butter, Vegetable, Earthy, Beany |
| 78 | Furaneol | Furans | 2033 | 2037 | 0.03 | 0.01 | 0.03 | 0.04 | 0.12 | 0.04 | Caramel, Strawberry, Grape |
| 79 | 2-Butanone | Ketones | 900 | 900 | 0.43 | 0.52 | 0.49 | 0.44 | 0.47 | 0.95 | Ether, Fruity, Acetone, Camphor |
| 80 | Isopropylideneacetone | Ketones | 1126 | 1125 | 1.30 | 1.16 | 1.75 | 1.88 | 3.13 | 1.59 | Vegetable, Earthy, Sweet |
| 81 | Butylacetone | Ketones | 1177 | 1180 | 0.03 | 0.04 | 0.02 | 0.02 | 0.02 | 0.01 | Coconut, Soap, Herbal, Sweet |
| 82 | Acetoin | Ketones | 1276 | 1280 | 0.46 | 0.31 | 0.43 | 0.50 | 0.68 | 0.42 | Butter, Milky, Fatty, Creamy |
| 83 | 2-Octanone | Ketones | 1280 | 1283 | 0.51 | 0.47 | 0.41 | 0.41 | 0.36 | 0.34 | Earthy, Gasoline, Weedy, Herbal |
| 84 | Hydroxyacetone | Ketones | 1290 | 1290 | 0.78 | 0.69 | 1.05 | 1.27 | 1.49 | 1.31 | Sweet, Pungent |
| 85 | 2,6,6-Trimethylcyclohexanone | Ketones | 1302 | 1282 | 0.27 | 0.38 | 0.49 | 0.48 | 0.59 | 1.08 | Pungent, Honey, Cistus |
| 86 | 2,3-Octanedione | Ketones | 1321 | 1325 | 0.27 | 0.22 | 0.34 | 0.46 | 0.40 | 0.20 | Buttery, Broccoli, Cooked, Green |
| 87 | Prenylacetone | Ketones | 1333 | 1339 | 0.34 | 0.42 | 0.34 | 0.33 | 0.32 | 0.36 | Apple, Mushroom, Citrus, Musty |
| 88 | Diacetone alcohol | Ketones | 1356 | 1352 | 0.11 | 0.13 | 0.33 | 0.17 | 0.23 | 0.26 | Faint pleasant, Minty |
| 89 | Isophorone | Ketones | 1586 | 1577 | 2.11 | 2.19 | 1.81 | 1.50 | 1.71 | 1.55 | Cooling, Tobacco, Cedarwood |
| 90 | Acetophenone | Ketones | 1642 | 1652 | 0.03 | 0.05 | 0.05 | 0.03 | 0.03 | 0.05 | Acacia, Almond, Hawthorne |
| 91 | Geranyl acetone | Ketones | 1852 | 1859 | 0.02 | 0.02 | 0.02 | 0.02 | 0.02 | 0.03 | Magnolia, Green |
| 92 | Jasmone | Ketones | 1939 | 1923 | 0.06 | 0.08 | 0.08 | 0.09 | 0.03 | 0.03 | Jasmine, Herbal, Floral |
| 93 | Perhydrofarnesyl acetone | Ketones | 2125 | 2110 | 0.02 | 0.01 | 0.01 | 0.01 | 0.01 | tr | Jasmin, Herbal, Fatty |
| 94 | [1,3-Diphenylpropanetrione](https://pubchem.ncbi.nlm.nih.gov/compound/223850) | Ketones | 2436 |  | tr | nd | nd | tr | nd | tr |  |
| 95 | Butyrolactone | Lactones | 1616 | 1617 | 0.07 | 0.07 | 0.07 | 0.09 | 0.10 | 0.03 | Fatty, Sweet, Creamy |
| 96 | 4-Methyl-4-vinylbutyrolactone | Lactones | 1660 | 1669 | 0.08 | 0.05 | 0.03 | 0.02 | 0.04 | 0.05 | Minty, Fruity, Fruity |
| 97 | 2-Penten-4-olide | Lactones | 1667 | 1664 | 0.01 | 0.02 | 0.02 | 0.02 | 0.01 | 0.02 | Solvent, Tobacco, Coumarin, Oily |
| 98 | Butenolide | Lactones | 1742 | 1745 | 0.01 | 0.02 | 0.02 | 0.03 | 0.03 | 0.02 | Buttery, Roasted |
| 99 | Dihydroactinidiolide | Lactones | 2318 | 2315 | 0.11 | 0.18 | 0.21 | 0.20 | 0.20 | 0.39 |  |
| 100 | Cyclofenchene | Monoterpenes | 1016 |  | 0.14 | 0.18 | 1.22 | 0.16 | 0.52 | 0.24 |  |
| 101 | Limonene | Monoterpenes | 1191 | 1189 | 1.12 | 0.43 | 0.75 | 0.21 | 0.39 | 1.28 | Lemon, Orange |
| 102 | 1,3,8-*p*-Menthatriene | Monoterpenes | 1318 |  | 0.28 | 0.28 | 0.28 | 0.31 | 0.21 | 0.22 | Herbal, Turpentine, Camphor |
| 103 | Linalool | Monoterpenes | 1548 | 1552 | 3.40 | 0.53 | 0.58 | 0.15 | 1.45 | 0.29 | Lemon, Citrus, Orange, Floral |
| 104 | Hotrienol | Monoterpenes | 1609 | 1613 | 0.12 | 0.03 | 0.03 | 0.02 | 0.19 | 0.27 | Hyacinth |
| 105 | *β*-Cyclocitral | Monoterpenes | 1615 | 1624 | 0.16 | 0.21 | 0.25 | 0.26 | 0.34 | 0.40 | Mint, Saffron, Tobacco |
| 106 | Safranal | Monoterpenes | 1639 |  | 0.04 | 0.04 | 0.06 | 0.06 | 0.08 | 0.07 | Spicy, Tobacco, Herbal, Fresh |
| 107 | Linderol | Monoterpenes | 1700 | 1675 | nd | nd | 0.04 | nd | nd | tr |  |
| 108 | *cis*-Linalool 3,7-oxide | Monoterpenes | 1763 | 1760 | 1.25 | 0.44 | 0.40 | 0.13 | 0.74 | 0.55 |  |
| 109 | Geraniol | Monoterpenes | 1848 | 1851 | 2.54 | 0.21 | 0.31 | 0.75 | 0.48 | 0.06 | Rose, Geranium |
| 110 | 4-Cyanocyclohexene | Nitriles | 1554 |  | 0.01 | 0.01 | 0.01 | 0.01 | 0.01 | 0.01 |  |
| 111 | Benzyl nitrile | Nitriles | 1917 | 1927 | 0.01 | nd | nd | nd | 0.04 | 0.03 | Aromatic |
| 112 | 2-Ethylpyrrole | Nitrogen heterocycles | 1174 |  | 0.33 | 0.33 | 0.59 | 0.84 | 0.79 | 0.42 |  |
| 113 | 2-Ethyl-3,6-dimethylpyrazine | Nitrogen heterocycles | 1443 | 1447 | tr | 0.01 | 0.03 | 0.07 | 0.06 | 0.01 | Roasted, Potato, Cocoa, Nutty |
| 114 | Pyrrole | Nitrogen heterocycles | 1509 | 1513 | 0.09 | 0.09 | 0.11 | 0.11 | 0.14 | 0.14 | Ethereal, Nutty, Sweet |
| 115 | 5-Methylpyrazole | Nitrogen heterocycles | 1777 |  | 0.09 | 0.15 | 0.14 | 0.13 | 0.09 | 0.09 |  |
| 116 | 2-Acetylpyrrole | Nitrogen heterocycles | 1964 | 1970 | 0.04 | 0.03 | 0.06 | 0.08 | 0.14 | 0.07 | Bread, Musty, Nutty, Walnut |
| 117 | 2-Formylpyrrole | Nitrogen heterocycles | 2016 | 2009 | tr | tr | 0.01 | 0.01 | 0.01 | 0.01 | Ethereal, Bread, Burnt, Caramel |
| 118 | Ethylmethylmaleimide | Nitrogen heterocycles | 2257 | 2260 | 0.01 | 0.01 | 0.01 | 0.01 | 0.01 | 0.01 |  |
| 119 | Methylvinylmaleimide | Nitrogen heterocycles | 2379 |  | 0.01 | 0.03 | 0.02 | 0.02 | 0.02 | 0.02 |  |
| 120 | Indole | Nitrogen heterocycles | 2405 | 2412 | 0.04 | 0.04 | 0.11 | 0.14 | 0.13 | 0.06 | Burnt, Jasmine, Fishy, Floral |
| 121 | 3-Methyl-4-aminophenol | Phenols | 1601 |  | 0.22 | 0.13 | 0.21 | 0.29 | 0.31 | 0.15 |  |
| 122 | Voidox | Phenols | 1892 |  | 0.15 | 0.07 | 0.21 | 0.08 | 0.17 | 0.09 |  |
| 123 | Butylated Hydroxytoluene | Phenols | 1909 | 1906 | 1.54 | 0.05 | 0.08 | 0.12 | 0.42 | 0.05 | Mild, Vanilla, Musty, Camphor |
| 124 | Phenol | Phenols | 1998 | 1992 | 0.05 | 0.07 | 0.12 | 0.11 | 0.14 | 0.08 | Phenol, Plastic, Rubber, Phenolic |
| 125 | Olivetol | Phenols | 2145 |  | 0.26 | 0.24 | 0.26 | 0.37 | 0.21 | 0.25 |  |
| 126 | 3-*tert*-Butylphenol | Phenols | 2276 |  | 0.05 | 0.04 | 0.09 | 0.05 | 0.10 | 0.04 |  |
| 127 | 2,4-Di-*tert*-butylphenol | Phenols | 2300 | 2321 | 0.15 | 0.12 | 0.12 | 0.12 | 0.12 | 0.12 |  |
| 128 | Copaene | Sesquiterpenes | 1487 | 1488 | 0.23 | 0.07 | 0.06 | 0.01 | 0.06 | 0.08 | Wood, Spice |
| 129 | Caryophyllene | Sesquiterpenes | 1591 | 1595 | 0.10 | 0.07 | 0.08 | 0.08 | 0.06 | 0.09 | Wood, Spice |
| 130 | *trans*-Calamenene | Sesquiterpenes | 1828 | 1844 | 0.02 | 0.01 | tr | nd | tr | 0.01 |  |
| 131 | *α*-Calacorene | Sesquiterpenes | 1910 | 1915 | 0.01 | tr | tr | nd | nd | tr | Wood |
| 132 | *α*-Nerolidol | Sesquiterpenes | 2040 | 2042 | 0.05 | 0.04 | 0.03 | 0.01 | 0.06 | 0.03 |  |
| 133 | T-Cadinol | Sesquiterpenes | 2168 | 2175 | tr | tr | tr | tr | tr | 0.01 |  |
| 134 | Dimethyl Sulfoxide | Others | 1570 | 1579 | 1.79 | 1.43 | 1.17 | 0.73 | 2.24 | 1.47 | Fatty, Mushroom, Cheesy, Garlic |
| 135 | Isovaleric acid | Others | 1713 | 1694 | tr | tr | nd | nd | nd | nd | Sour, Sweat, Acid, Stinky, Sweat |
| 136 | Dimethyl sulfone | Others | 1888 | 1895 | 0.08 | 0.07 | 0.05 | 0.03 | 0.07 | 0.07 | Burnt, Sulfurous |
| 137 | Tetramethyl decynediol | Others | 2100 |  | 0.02 | 0.01 | 0.01 | 0.01 | 0.01 | 0.01 |  |

*^a^* Volatile compounds were characterized by comparing their linear retention indices and MS spectra with those in NIST 23 database.

*^b^* LRI calculated on a DB-Wax column using C_7_-C_30_ *n*-alkanes as reference standards.

*^c^* Reference LRI on a DB-Wax column retrieved from the NIST Chemistry WebBook.

*^d^* nd: not detected, tr: trace compounds (< 0.01%).

**Table S2.** Non-volatiles characterized from GT with different grades using UPLC-QE-Orbitrap-HRMS-based metabolomics.

| No. | Compound annotation | RT (s) *^a^* | *m/z* | Formula | Class*^b^* | Mode*^C^* | *S*_total_*^d^* | *S_m/z_^e^* | *S*_RT_*^f^* | *S*_MS/MS_*^g^* | IS*^h^* |
| --- | --- | --- | --- | --- | --- | --- | --- | --- | --- | --- | --- |
| 1 | Pyrrolidine | 45.3 | 72.0808 | C₄H₉N | A | P | 3.91 | 0.97 | 0.66 | 1.00 | L1 |
| 2 | *N*-Acetylputrescine | 45.8 | 131.1178 | C₆H₁₄N₂O | A | P | 3.83 | 0.95 | 0.58 | 0.90 | L1 |
| 3 | 7-Methylxanthine | 103.7 | 167.0563 | C₆H₆N₄O₂ | A | P/N | 3.92 | 0.99 | 0.71 | 1.00 | L1 |
| 4 | 2-Piperidone | 111.0 | 100.0757 | C₅H₉NO | A | P | 3.95 | 0.96 | 0.86 | 1.00 | L1 |
| 5 | 3-Methylxanthine | 114.5 | 167.0563 | C₆H₆N₄O₂ | A | P/N | 3.94 | 0.99 | 0.80 | 1.00 | L1 |
| 6 | 3,7-Dimethyluric acid | 135.2 | 195.0523 | C₇H₈N₄O₃ | A | N | 3.87 | 1.00 | 0.50 | 1.00 | L1 |
| 7 | Theobromine | 201.2 | 181.0720 | C₇H₈N₄O₂ | A | P/N | 3.91 | 0.97 | 0.71 | 0.97 | L1 |
| 8 | Isoquinoline | 204.8 | 130.0656 | C₉H₇N | A | P | 3.94 | 0.77 | 0.99 | 1.00 | L1 |
| 9 | Theophylline | 208.9 | 181.0716 | C₇H₈N₄O₂ | A | P/N | 3.96 | 0.91 | 0.93 | 1.00 | L1 |
| 10 | *N*-Feruloylputrescine | 213.0 | 265.1546 | C₁₄H₂₀N₂O₃ | A | P | 3.89 | 0.98 | 0.59 | 0.99 | L1 |
| 11 | 5-Hydroxyindole | 229.1 | 134.0600 | C₈H₇NO | A | P | 3.95 | 0.97 | 0.86 | 0.99 | L1 |
| 12 | Caffeine | 242.5 | 195.0872 | C₈H₁₀N₄O₂ | A | P | 3.81 | 0.83 | 0.52 | 0.99 | L1 |
| 13 | 2-Oxindole-3-acetate / Methyl 5-hydroxy-3-indolecarboxylate | 268.0 | 190.0508 | C₁₀H₉NO₃ | A | N | 3.86–3.95 | 0.94 | 0.56–0.93 | 0.96–0.97 | UI |
| 14 | Indole-3-carboxaldehyde | 312.8 | 146.0599 | C₉H₇NO | A | P/N | 3.88 | 0.96 | 0.59 | 0.99 | L1 |
| 15 | Pipecolamide | 46.6 | 129.1022 | C₆H₁₂N₂O | B | P | 3.86 | 0.97 | 0.61 | 0.93 | L1 |
| 16 | NAE(18:3(6*Z*,9*Z*,12*Z*)) / NAE(18:3(9*Z*,12*Z*,15*Z*)) | 502.6 | 322.2739 | C₂₀H₃₅NO₂ | B | P | 3.90–3.91 | 0.96 | 0.74–0.84 | 0.92–0.95 | UI |
| 17 | NAE(14:0) | 507.3 | 272.2583 | C₁₆H₃₃NO₂ | B | P | 3.86 | 0.96 | 0.59 | 0.94 | L1 |
| 18 | Linoleamide | 526.9 | 280.2629 | C₁₈H₃₃NO | B | P | 3.80 | 0.86 | 0.50 | 0.91 | L1 |
| 19 | Glycine | 28.3 | 76.0393 | C₂H₅NO₂ | C | P/N | 3.88 | 0.98 | 0.55 | 1.00 | L1 |
| 20 | Lysine | 38.8 | 147.1128 | C₆H₁₄N₂O₂ | C | P/N | 3.90 | 0.98 | 0.63 | 0.99 | L1 |
| 21 | *β*-Alanine | 41.3 | 88.0403 | C₃H₇NO₂ | C | N | 3.91 | 0.94 | 0.71 | 1.00 | L1 |
| 22 | 3-Ureidopropionic acid / Asparagine / Diglycine | 41.4 | 131.0461 | C₄H₈N₂O₃ | C | N | 3.84–3.90 | 0.97 | 0.74–0.86 | 0.80–0.94 | UI |
| 23 | Arginine | 41.4 | 173.1043 | C₆H₁₄N₄O₂ | C | N | 3.92 | 0.99 | 0.69 | 1.00 | L1 |
| 24 | 4-Aminobutyric acid | 41.6 | 102.0559 | C₄H₉NO₂ | C | N | 3.91 | 0.95 | 0.70 | 1.00 | L1 |
| 25 | Ala-Gly | 41.8 | 145.0618 | C₅H₁₀N₂O₃ | C | N | 3.95 | 0.97 | 0.89 | 0.97 | L1 |
| 26 | Homoserine | 42.1 | 118.0509 | C₄H₉NO₃ | C | N | 3.92 | 0.97 | 0.74 | 0.98 | L1 |
| 27 | Histidine | 42.2 | 156.0767 | C₆H₉N₃O₂ | C | P/N | 3.82 | 0.97 | 0.53 | 0.90 | L1 |
| 28 | Sarcosine | 42.6 | 90.0549 | C₃H₇NO₂ | C | P/N | 3.92 | 0.93 | 0.73 | 1.00 | L1 |
| 29 | Glutamine | 42.8 | 147.0763 | C₅H₁₀N₂O₃ | C | P/N | 3.87 | 0.96 | 0.71 | 0.90 | L1 |
| 30 | Serine | 42.9 | 106.0498 | C₃H₇NO₃ | C | P/N | 3.90 | 0.94 | 0.70 | 0.98 | L1 |
| 31 | Glutamate | 43.0 | 148.0603 | C₅H₉NO₄ | C | P/N | 3.94 | 0.95 | 0.84 | 0.98 | L1 |
| 32 | L-Homoarginine | 43.3 | 189.1345 | C₇H₁₆N₄O₂ | C | P | 3.88 | 0.95 | 0.67 | 0.94 | L1 |
| 33 | Proline | 43.6 | 116.0705 | C₅H₉NO₂ | C | P/N | 3.92 | 0.95 | 0.72 | 0.99 | L1 |
| 34 | Stachydrine | 45.1 | 144.1018 | C₇H₁₃NO₂ | C | P | 3.93 | 0.96 | 0.77 | 1.00 | L1 |
| 35 | Betaine / *α*-(Methylamino)isobutyric acid | 45.3 | 118.0862 | C₅H₁₁NO₂ | C | P | 3.90–3.92 | 0.95 | 0.64–0.76 | 0.98–1.00 | UI |
| 36 | Hygric acid / Pipecolic acid | 45.3 | 130.0862 | C₆H₁₁NO₂ | C | P | 3.93–3.96 | 0.97 | 0.80–0.86 | 0.98–1.00 | UI |
| 37 | Valine | 45.7 | 118.0862 | C₅H₁₁NO₂ | C | P/N | 3.92 | 0.96 | 0.75 | 0.99 | L1 |
| 38 | L-Valyl-L-alanine | 45.9 | 189.1236 | C₈H₁₆N₂O₃ | C | P | 3.89 | 0.93 | 0.83 | 0.90 | L1 |
| 39 | Pyroglutamic acid | 46.6 | 130.0498 | C₅H₇NO₃ | C | P/N | 3.86 | 0.96 | 0.53 | 0.98 | L1 |
| 40 | 6-Hydroxynorleucine | 46.8 | 128.0715 | C₆H₁₃NO₃ | C | N | 3.90 | 0.94 | 0.65 | 1.00 | L1 |
| 41 | 2-Aminoadipic acid | 47.5 | 160.0614 | C₆H₁₁NO₄ | C | N | 3.87 | 0.96 | 0.69 | 0.92 | L1 |
| 42 | Theanine | 52.8 | 175.1074 | C₇H₁₄N₂O₃ | C | P/N | 3.84 | 0.88 | 0.57 | 0.95 | L1 |
| 43 | *N*-Acetyl-arginine | 54.5 | 217.1298 | C₈H₁₆N₄O₃ | C | P | 3.91 | 0.93 | 0.81 | 0.95 | L1 |
| 44 | *N*-Acetylglutamic acid | 58.6 | 188.0564 | C₇H₁₁NO₅ | C | N | 3.87 | 0.97 | 0.77 | 0.87 | L1 |
| 45 | Tyrosine | 59.2 | 182.0811 | C₉H₁₁NO₃ | C | P/N | 3.88 | 0.96 | 0.63 | 0.97 | L1 |
| 46 | *N*-Acetylalanine / *N*-Acetyl-*β*-alanine | 59.4 | 130.0509 | C₅H₉NO₃ | C | N | 3.89–3.93 | 0.97 | 0.73–1.00 | 0.88–0.93 | UI |
| 47 | Leucine | 63.2 | 132.1017 | C₆H₁₃NO₂ | C | P/N | 3.92 | 0.93 | 0.76 | 1.00 | L1 |
| 48 | 5-Hydroxytryptophan | 72.7 | 219.0774 | C₁₁H₁₂N₂O₃ | C | N | 3.86 | 0.97 | 0.74 | 0.87 | L1 |
| 49 | 5-Oxo-L-prolyl-L-proline | 97.2 | 225.0880 | C₁₀H₁₄N₂O₄ | C | N | 3.95 | 0.99 | 0.96 | 0.93 | L1 |
| 50 | Phenylalanine | 100.7 | 166.0859 | C₉H₁₁NO₂ | C | P/N | 3.89 | 0.90 | 0.70 | 0.98 | L1 |
| 51 | Methyl pyroglutamate | 104.4 | 144.0656 | C₆H₉NO₃ | C | P | 3.97 | 0.99 | 0.90 | 1.00 | L1 |
| 52 | Aspartylphenylalanine | 191.0 | 281.1141 | C₁₃H₁₆N₂O₅ | C | P | 3.81 | 0.80 | 0.78 | 0.82 | L1 |
| 53 | Tryptophan | 206.3 | 205.0969 | C₁₁H₁₂N₂O₂ | C | P/N | 3.82 | 0.92 | 0.54 | 0.96 | L1 |
| 54 | *N*-Acetyltryptophan | 274.6 | 245.0930 | C₁₃H₁₄N₂O₃ | C | N | 3.87 | 0.95 | 0.68 | 0.91 | L1 |
| 55 | *N*-Formyltryptophan | 277.9 | 231.0773 | C₁₂H₁₂N₂O₃ | C | N | 3.85 | 0.95 | 0.61 | 0.91 | L1 |
| 56 | Gentisic acid 5-*O*-*β*-glucoside | 97.9 | 315.0719 | C₁₃H₁₆O₉ | D | N | 3.93 | 0.95 | 0.98 | 0.90 | L1 |
| 57 | 4-Hydroxybenzoic acid | 218.3 | 137.0243 | C₇H₆O₃ | D | N | 3.86 | 0.97 | 0.56 | 0.96 | L1 |
| 58 | Corilagin | 235.0 | 633.0719 | C₂₇H₂₂O₁₈ | D | N | 3.90 | 0.85 | 0.88 | 0.93 | L1 |
| 59 | 2-Acetylbenzoic acid | 237.1 | 163.0399 | C₉H₈O₃ | D | N | 3.86 | 0.96 | 0.55 | 0.97 | L1 |
| 60 | Methylgallate | 246.0 | 183.0296 | C₈H₈O₅ | D | N | 3.89 | 0.92 | 0.76 | 0.93 | L1 |
| 61 | 3-Hydroxybenzoic acid | 249.5 | 137.0243 | C₇H₆O₃ | D | N | 3.96 | 0.97 | 0.93 | 0.97 | L1 |
| 62 | 3-Carboxymethylbenzoic acid | 265.5 | 179.0348 | C₉H₈O₄ | D | N | 3.90 | 0.95 | 0.79 | 0.92 | L1 |
| 63 | Tropic acid | 266.0 | 147.0451 | C₉H₁₀O₃ | D | N | 3.87 | 0.99 | 0.57 | 0.96 | L1 |
| 64 | 2,6-Dihydroxybenzoic acid | 266.2 | 153.0192 | C₇H₆O₄ | D | N | 3.86 | 0.95 | 0.54 | 0.98 | L1 |
| 65 | 2-Phenyllactic acid / Desaminotyrosine | 270.2 | 165.0556 | C₉H₁₀O₃ | D | N | 3.82–3.89 | 0.95 | 0.59–0.67 | 0.86–0.97 | UI |
| 66 | Salicylic acid | 295.9 | 137.0243 | C₇H₆O₃ | D | N | 3.89 | 0.95 | 0.83 | 0.90 | L1 |
| 67 | Piperonylic acid | 313.6 | 165.0193 | C₈H₆O₄ | D | N | 3.93 | 0.98 | 0.90 | 0.92 | L1 |
| 68 | Dimethyl 4-hydroxyisophthalate | 368.8 | 209.0454 | C₁₀H₁₀O₅ | D | N | 3.85 | 0.96 | 0.58 | 0.93 | L1 |
| 69 | Ellagic acid | 523.4 | 300.9989 | C₁₄H₆O₈ | D | N | 3.94 | 0.97 | 0.80 | 1.00 | L1 |
| 70 | Fructose 1-phosphate / Fructose 6-phosphate /  Mannose 6-phosphate | 39.5 | 259.0224 | C₆H₁₃O₉P | E | N | 3.87–3.90 | 0.99 | 0.67–0.71 | 0.91–0.96 | UI |
| 71 | Threonic acid | 40.5 | 135.0298 | C₄H₈O₅ | E | N | 3.96 | 0.97 | 0.91 | 0.99 | L1 |
| 72 | Gluconic acid | 40.8 | 195.0509 | C₆H₁₂O₇ | E | N | 3.93 | 0.98 | 0.96 | 0.90 | L1 |
| 73 | D-Mannoheptulose | 42.9 | 209.0665 | C₇H₁₄O₇ | E | N | 3.92 | 0.97 | 0.77 | 0.96 | L1 |
| 74 | Glucose 1-phosphate | 43.3 | 261.0369 | C₆H₁₃O₉P | E | P | 3.90 | 0.97 | 0.78 | 0.92 | L1 |
| 75 | Isomaltose / Gentiobiose / Trehalose / Palatinose /  Galactinol / Neotrehalose / Turanose / Sucrose | 44.2 | 341.1084 | C₁₂H₂₂O₁₁ | E | N | 3.84–3.91 | 0.89 | 0.68–0.80 | 0.88–0.97 | UI |
| 76 | 2-*O*-(*β*-D-Glucopyranosyl)ascorbic acid | 44.9 | 337.0774 | C₁₂H₁₈O₁₁ | E | N | 3.97 | 0.96 | 0.95 | 1.00 | L1 |
| 77 | Raffinose | 45.8 | 527.1588 | C₁₈H₃₂O₁₆ | E | P | 3.93 | 0.94 | 0.77 | 1.00 | L1 |
| 78 | Arabinono-1,4-lactone | 50.5 | 147.0298 | C₅H₈O₅ | E | N | 3.82 | 0.97 | 0.59 | 0.85 | L1 |
| 79 | Glutaric acid | 101.2 | 131.0349 | C₅H₈O₄ | E | N | 3.94 | 0.99 | 0.81 | 0.98 | L1 |
| 80 | Undulatoside A | 234.0 | 355.1021 | C₁₆H₁₈O₉ | F | P | 3.95 | 0.95 | 0.84 | 1.00 | L1 |
| 81 | 5,7-Dihydroxychromone | 287.9 | 177.0192 | C₉H₆O₄ | F | N | 3.86 | 0.95 | 0.73 | 0.87 | L1 |
| 82 | Butyryl-L-carnitine / Isobutyrylcarnitine | 99.6 | 232.1543 | C₁₁H₂₁NO₄ | G | P | 3.81–3.85 | 0.98 | 0.51–0.62 | 0.89–0.90 | UI |
| 83 | Butyllactate | 331.4 | 145.0870 | C₇H₁₄O₃ | G | N | 3.85 | 0.98 | 0.62 | 0.91 | L1 |
| 84 | Methyl 2-pentenyl-3-oxocyclopentaneacetate | 401.2 | 225.1484 | C₁₃H₂₀O₃ | G | P | 3.92 | 0.96 | 0.81 | 0.96 | L1 |
| 85 | Glyceric acid | 43.7 | 105.0192 | C₃H₆O₄ | H | N | 3.98 | 0.95 | 0.99 | 0.99 | L1 |
| 86 | 2,4-Dihydroxybutanoic acid | 44.2 | 119.0349 | C₄H₈O₄ | H | N | 3.91 | 0.97 | 0.86 | 0.91 | L1 |
| 87 | 3-Hydroxypyruvic acid / Malonic acid | 45.5 | 103.0036 | C₃H₄O₄ | H | N | 3.88 | 0.94 | 0.58–0.87 | 0.86–0.99 | UI |
| 88 | 2-Methylcitrate | 48.2 | 205.0353 | C₇H₁₀O₇ | H | N | 3.88 | 0.98 | 0.70 | 0.93 | L1 |
| 89 | 2-Hydroxybutyric acid / 2-Hydroxyisobutyric acid /  3-Hydroxybutyric acid | 68.6 | 103.0400 | C₄H₈O₃ | H | N | 3.89–3.96 | 0.96 | 0.71–0.98 | 0.91–0.94 | UI |
| 90 | 3-Butynoic acid | 68.8 | 85.0284 | C₄H₄O₂ | H | P | 3.96 | 0.94 | 0.90 | 1.00 | L1 |
| 91 | 4-Acetamidobutyric acid | 70.6 | 144.0665 | C₆H₁₁NO₃ | H | N | 3.85 | 0.98 | 0.78 | 0.82 | L1 |
| 92 | Mesaconic acid | 74.4 | 129.0192 | C₅H₆O₄ | H | N | 3.96 | 0.97 | 0.89 | 1.00 | L1 |
| 93 | Levulinic acid | 78.6 | 115.0400 | C₅H₈O₃ | H | N | 3.93 | 0.95 | 0.86 | 0.96 | L1 |
| 94 | 2-Hydroxyhexanedioic acid / 3-Hydroxy-3-methylglutaric acid | 78.8 | 161.0455 | C₆H₁₀O₅ | H | N | 3.88–3.97 | 0.97 | 0.91–0.98 | 0.82–0.96 | UI |
| 95 | Maleic acid | 91.1 | 115.0036 | C₄H₄O₄ | H | N | 3.90 | 0.98 | 0.63 | 1.00 | L1 |
| 96 | 2-Aminoheptanoic acid | 118.3 | 144.1030 | C₇H₁₅NO₂ | H | N | 3.89 | 0.99 | 0.56 | 1.00 | L1 |
| 97 | Ethylmalonic acid / Methylsuccinic acid | 125.3 | 131.0350 | C₅H₈O₄ | H | N | 3.87–3.91 | 0.99 | 0.56–0.79 | 0.92–0.96 | UI |
| 98 | 3-Hydroxyvaleric acid | 139.2 | 99.0451 | C₅H₁₀O₃ | H | N | 3.96 | 0.99 | 0.85 | 1.00 | L1 |
| 99 | 2-Hydroxy-2-methylbutyric acid | 139.6 | 117.0557 | C₅H₁₀O₃ | H | N | 3.93 | 0.99 | 0.72 | 1.00 | L1 |
| 100 | 4-Oxohexanoic acid | 199.0 | 129.0557 | C₆H₁₀O₃ | H | N | 3.89 | 0.99 | 0.83 | 0.87 | L1 |
| 101 | 2-Isopropylmalic acid / 3-Isopropylmalic acid | 213.6 | 175.0610 | C₇H₁₂O₅ | H | N | 3.89–3.93 | 0.95 | 0.77–0.83 | 0.91–0.98 | UI |
| 102 | 2-Methylglutaric acid / 3-Methylglutaric acid / Adipic acid | 214.1 | 145.0506 | C₆H₁₀O₄ | H | N | 3.81–3.87 | 0.97 | 0.53–0.73 | 0.86–0.98 | UI |
| 103 | 2-Ethyl-2-hydroxybutyric acid | 215.2 | 131.0713 | C₆H₁₂O₃ | H | N | 3.91 | 0.97 | 0.69 | 1.00 | L1 |
| 104 | 2-Hydroxyhexanoic acid / Hydroxyisocaproic acid | 256.1 | 131.0713 | C₆H₁₂O₃ | H | N | 3.95–3.97 | 0.96 | 0.87–0.92 | 0.98–1.00 | UI |
| 105 | 1-Hydroxycyclohexaneacetic acid / 5-Oxooctanoic acid | 279.9 | 157.0869 | C₈H₁₄O₃ | H | N | 3.85–3.94 | 0.97 | 0.72–0.89 | 0.86–0.95 | UI |
| 106 | Suberic acid | 293.6 | 173.0818 | C₈H₁₄O₄ | H | N | 3.85 | 0.96 | 0.56 | 0.94 | L1 |
| 107 | 5-Hydroxyvalproic acid | 298.8 | 159.1026 | C₈H₁₆O₃ | H | N | 3.99 | 0.97 | 0.99 | 0.99 | L1 |
| 108 | Isovaleric acid / Valeric acid | 305.7 | 101.0607 | C₅H₁₀O₂ | H | N | 3.86–3.95 | 0.94 | 0.50–0.86 | 1.00 | UI |
| 109 | Azelaic acid | 327.0 | 187.0975 | C₉H₁₆O₄ | H | N | 3.88 | 0.99 | 0.62 | 0.95 | L1 |
| 110 | Royal jelly acid | 344.3 | 185.1182 | C₁₀H₁₈O₃ | H | N | 3.97 | 0.98 | 0.89 | 1.00 | L1 |
| 111 | 3-Hydroxyoctanoic acid | 352.3 | 159.1026 | C₈H₁₆O₃ | H | N | 3.91 | 0.97 | 0.74 | 0.97 | L1 |
| 112 | Sebacic acid | 355.0 | 201.1132 | C₁₀H₁₈O₄ | H | N | 3.88 | 0.98 | 0.64 | 0.95 | L1 |
| 113 | 10-Hydroxydecanoic acid | 360.4 | 187.1339 | C₁₀H₂₀O₃ | H | N | 3.87 | 0.98 | 0.67 | 0.92 | L1 |
| 114 | (*E*)-4-Hydroxynon-2-enoic acid | 364.2 | 171.1026 | C₉H₁₆O₃ | H | N | 3.83 | 0.97 | 0.54 | 0.91 | L1 |
| 115 | Caproic acid | 365.6 | 115.0764 | C₆H₁₂O₂ | H | N | 3.90 | 0.96 | 0.63 | 1.00 | L1 |
| 116 | (4*E*,7*E*)-Dodeca-4,7-dienedioic acid | 370.1 | 225.1131 | C₁₂H₁₈O₄ | H | N | 3.81 | 0.98 | 0.60 | 0.84 | L1 |
| 117 | Traumatic acid | 386.4 | 227.1288 | C₁₂H₂₀O₄ | H | N | 3.94 | 0.99 | 0.84 | 0.98 | L1 |
| 118 | 9,12,13-Trihydroxy-10*E*-octadecenoic acid /  Pinellic acid / (*Z*)-9,12,13-trihydroxyoctadec-15-enoic acid | 394.0 | 329.2333 | C₁₈H₃₄O₅ | H | N | 3.88–3.89 | 1.00 | 0.62–0.67 | 0.92–0.97 | UI |
| 119 | Dodecanedioic acid | 400.2 | 229.1445 | C₁₂H₂₂O₄ | H | N | 3.96 | 0.99 | 0.95 | 0.95 | L1 |
| 120 | 2-Methylcaproic acid | 401.6 | 129.0920 | C₇H₁₄O₂ | H | N | 3.88 | 0.98 | 0.53 | 1.00 | L1 |
| 121 | *trans*-2-Octenoic acid | 417.4 | 141.0920 | C₈H₁₄O₂ | H | N | 3.95 | 0.98 | 0.83 | 1.00 | L1 |
| 122 | 2-Methylheptanoic acid / Caprylic acid | 429.9 | 143.1077 | C₈H₁₆O₂ | H | N | 3.91–3.95 | 0.97 | 0.69–0.85 | 1.00 | UI |
| 123 | Pelargonic acid | 454.1 | 157.1233 | C₉H₁₈O₂ | H | N | 3.98 | 0.96 | 0.95 | 1.00 | L1 |
| 124 | 4-Ethyloctanoic acid | 464.6 | 171.1390 | C₁₀H₂₀O₂ | H | N | 3.98 | 0.99 | 0.94 | 1.00 | L1 |
| 125 | (2*E*,4*Z*)-9-Oxooctadeca-2,4-dienoic acid | 466.6 | 293.2121 | C₁₈H₃₀O₃ | H | N | 3.81 | 0.97 | 0.53 | 0.88 | L1 |
| 126 | 13(*S*)-Hydroperoxylinolenic acid | 468.9 | 291.1965 | C₁₈H₃₀O₄ | H | N | 3.96 | 0.99 | 0.96 | 0.95 | L1 |
| 127 | 10-Undecenoic acid | 474.9 | 183.1390 | C₁₁H₂₀O₂ | H | N | 3.96 | 0.99 | 0.85 | 1.00 | L1 |
| 128 | 16-Hydroxypalmitic acid | 480.7 | 271.2278 | C₁₆H₃₂O₃ | H | N | 3.98 | 0.99 | 0.94 | 1.00 | L1 |
| 129 | (*S*)-Coriolic acid | 484.5 | 295.2278 | C₁₈H₃₂O₃ | H | N | 3.98 | 0.99 | 0.96 | 0.99 | L1 |
| 130 | (*E*)-11-Hydroxyoctadec-9-enoic acid /  (*E*)-6-Hydroxyoctadec-4-enoic acid | 495.1 | 297.2434 | C₁₈H₃₄O₃ | H | N | 3.90–3.95 | 0.99 | 0.84–0.86 | 0.87–1.00 | UI |
| 131 | Undecanoic acid | 495.1 | 185.1546 | C₁₁H₂₂O₂ | H | N | 3.98 | 0.99 | 0.95 | 1.00 | L1 |
| 132 | 9-Oxo-10(*E*),12(*E*)-octadecadienoic acid | 496.4 | 277.2160 | C₁₈H₃₀O₃ | H | P/N | 3.88 | 0.98 | 0.67 | 0.94 | L1 |
| 133 | Octadecanedioic acid | 502.5 | 313.2385 | C₁₈H₃₄O₄ | H | N | 3.92 | 0.99 | 0.93 | 0.87 | L1 |
| 134 | 9-Oxooctadecanoic acid | 507.6 | 281.2473 | C₁₈H₃₄O₃ | H | P/N | 3.89 | 0.97 | 0.76 | 0.92 | L1 |
| 135 | 12-Hydroxystearic acid | 511.8 | 299.2590 | C₁₈H₃₆O₃ | H | N | 3.92 | 0.97 | 0.72 | 1.00 | L1 |
| 136 | Myristoleic acid | 521.2 | 225.1860 | C₁₄H₂₆O₂ | H | N | 3.95 | 0.99 | 0.79 | 1.00 | L1 |
| 137 | Stearidonic acid | 525.5 | 275.2016 | C₁₈H₂₈O₂ | H | N | 3.93 | 0.99 | 0.72 | 1.00 | L1 |
| 138 | 2-Hydroxypalmitic acid | 529.7 | 271.2278 | C₁₆H₃₂O₃ | H | N | 3.89 | 0.99 | 0.59 | 0.99 | L1 |
| 139 | *α*-Linolenic acid | 534.3 | 277.2168 | C₁₈H₃₀O₂ | H | N | 3.97 | 0.88 | 0.99 | 1.00 | L1 |
| 140 | (*Z*)-2-Hydroxyoctadec-9-enoic acid | 535.6 | 297.2430 | C₁₈H₃₄O₃ | H | N | 3.85 | 0.90 | 0.61 | 0.94 | L1 |
| 141 | 12-Methyltridecanoic acid / Myristic acid | 543.0 | 227.2015 | C₁₄H₂₈O₂ | H | N | 3.95–3.98 | 0.95 | 0.83–0.96 | 1.00 | UI |
| 142 | *cis*-9-Palmitoleic acid | 546.4 | 253.2171 | C₁₆H₃₀O₂ | H | N | 3.98 | 0.96 | 0.96 | 1.00 | L1 |
| 143 | 17-Octadecynoic acid / Linoleic acid | 551.3 | 279.2326 | C₁₈H₃₂O₂ | H | N | 3.87–3.97 | 0.91 | 0.55–0.98 | 1.00 | UI |
| 144 | Pentadecanoic acid | 555.7 | 241.2172 | C₁₅H₃₀O₂ | H | N | 3.97 | 0.97 | 0.93 | 1.00 | L1 |
| 145 | Mead acid | 556.4 | 305.2485 | C₂₀H₃₄O₂ | H | N | 3.91 | 0.98 | 0.67 | 1.00 | L1 |
| 146 | 2-Hydroxystearic acid | 568.3 | 299.2589 | C₁₈H₃₆O₃ | H | N | 3.86 | 0.95 | 0.51 | 0.98 | L1 |
| 147 | Oleic acid | 569.7 | 281.2483 | C₁₈H₃₄O₂ | H | N | 3.97 | 0.94 | 0.95 | 1.00 | L1 |
| 148 | Tetracosanoic acid | 571.0 | 367.3578 | C₂₄H₄₈O₂ | H | N | 3.98 | 0.93 | 0.97 | 1.00 | L1 |
| 149 | Heptadecanoic acid | 579.4 | 269.2484 | C₁₇H₃₄O₂ | H | N | 3.95 | 0.95 | 0.85 | 1.00 | L1 |
| 150 | Paullinic acid / *trans*-11-Eicosenoic acid | 589.1 | 309.2797 | C₂₀H₃₈O₂ | H | N | 3.95–3.99 | 0.96 | 0.83–1.00 | 1.00 | UI |
| 151 | Stearic acid | 589.5 | 283.2641 | C₁₈H₃₆O₂ | H | N | 3.96 | 0.97 | 0.87 | 1.00 | L1 |
| 152 | Erucic acid | 608.4 | 337.3111 | C₂₂H₄₂O₂ | H | N | 3.96 | 0.99 | 0.87 | 1.00 | L1 |
| 153 | Phytanic acid | 610.0 | 311.2954 | C₂₀H₄₀O₂ | H | N | 3.87 | 0.97 | 0.50 | 1.00 | L1 |
| 154 | Nervonic acid | 629.5 | 365.3422 | C₂₄H₄₆O₂ | H | N | 3.97 | 0.94 | 0.93 | 1.00 | L1 |
| 155 | (+)-Gallocatechin | 212.8 | 307.0811 | C₁₅H₁₄O₇ | I | P/N | 3.87 | 0.93 | 0.72 | 0.92 | L1 |
| 156 | (-)-Catechin | 227.3 | 291.0862 | C₁₅H₁₄O₆ | I | P | 3.92 | 0.98 | 0.77 | 0.97 | L1 |
| 157 | Epigallocatechin | 235.3 | 307.0806 | C₁₅H₁₄O₇ | I | P/N | 3.85 | 0.88 | 0.70 | 0.91 | L1 |
| 158 | Leucocyanidin | 235.6 | 305.0662 | C₁₅H₁₄O₇ | I | N | 3.86 | 0.89 | 0.80 | 0.87 | L1 |
| 159 | Procyanidin B2 | 241.3 | 579.1491 | C₃₀H₂₆O₁₂ | I | P/N | 3.94 | 0.91 | 0.85 | 0.99 | L1 |
| 160 | Epicatechin | 242.6 | 291.0860 | C₁₅H₁₄O₆ | I | P | 3.91 | 0.93 | 0.80 | 0.96 | L1 |
| 161 | Swertiajaponin | 245.7 | 461.1082 | C₂₂H₂₂O₁₁ | I | N | 3.90 | 0.89 | 0.71 | 0.99 | L1 |
| 162 | Procyanidin C1 | 254.1 | 867.2130 | C₄₅H₃₈O₁₈ | I | P/N | 3.91 | 0.97 | 0.77 | 0.96 | L1 |
| 163 | Saponarin | 254.4 | 595.1654 | C₂₇H₃₀O₁₅ | I | P | 3.91 | 0.95 | 0.93 | 0.88 | L1 |
| 164 | Epigallocatechin gallate / Gallocatechin gallate | 258.7 | 459.0913 | C₂₂H₁₈O₁₁ | I | P/N | 3.90–3.95 | 0.85 | 0.75–0.97 | 1.00 | UI |
| 165 | Corymboside / Isoschaftoside / Schaftoside / Vicenin 3 | 267.2 | 565.1544 | C₂₆H₂₈O₁₄ | I | P | 3.86–3.95 | 0.90 | 0.58–0.97 | 0.97–0.98 | UI |
| 166 | 2′,3,5,6′,7-Pentahydroxyflavanone | 269.3 | 303.0506 | C₁₅H₁₂O₇ | I | N | 3.79 | 0.90 | 0.60 | 0.84 | L1 |
| 167 | Isoorientin | 273.1 | 449.1076 | C₂₁H₂₀O₁₁ | I | P | 3.90 | 0.95 | 0.67 | 0.99 | L1 |
| 168 | (-)-Epiafzelechin | 275.7 | 275.0912 | C₁₅H₁₄O₅ | I | P | 3.91 | 0.95 | 0.69 | 0.99 | L1 |
| 169 | Luteolin 7-*O*-rutinoside | 280.3 | 595.1653 | C₂₇H₃₀O₁₅ | I | P | 3.79 | 0.95 | 0.53 | 0.84 | L1 |
| 170 | Gossypin / Myricetin 3-*O*-galactoside / Quercetagitrin | 282.5 | 481.0972 | C₂₁H₂₀O₁₃ | I | P | 3.73–3.82 | 0.93 | 0.52-0.54 | 0.99-1.00 | UI |
| 171 | (-)-Catechin gallate | 282.7 | 443.0963 | C₂₂H₁₈O₁₀ | I | P | 3.90 | 0.85 | 0.86 | 0.94 | L1 |
| 172 | 2"-*O*-Galloylhyperin / Desmanthin 1 /  Quercetin 3-*O*-*β*-galactoside-6-gallate | 286.9 | 617.1132 | C₂₈H₂₄O₁₆ | I | P | 3.86–3.92 | 0.94 | 0.58–0.98 | 0.86–0.99 | UI |
| 173 | Kaempferol 3-neohesperidoside /  Kaempferol 7-*O*-neohesperidoside | 287.8 | 595.1653 | C₂₇H₃₀O₁₅ | I | P/N | 3.78–3.81 | 0.72 | 0.65–0.76 | 0.87–0.88 | UI |
| 174 | Plantagoside | 288.5 | 465.1036 | C₂₁H₂₂O₁₂ | I | N | 3.95 | 0.96 | 0.93 | 0.95 | L1 |
| 175 | Quercetin-3′-*β*-D-glucopyranosyl-(1→2)-*α*-L-rhamnopyranoside | 292.3 | 609.1453 | C₂₇H₃₀O₁₆ | I | N | 3.84 | 0.91 | 0.77 | 0.83 | L1 |
| 176 | Rutin | 292.3 | 611.1603 | C₂₇H₃₀O₁₆ | I | P/N | 3.84 | 0.94 | 0.54 | 0.99 | L1 |
| 177 | Kaempferol 7-*O*-glucoside | 294.9 | 449.1076 | C₂₁H₂₀O₁₁ | I | P | 3.94 | 0.95 | 0.83 | 1.00 | L1 |
| 178 | Apiin | 298.2 | 565.1546 | C₂₆H₂₈O₁₄ | I | P | 3.87 | 0.93 | 0.54 | 1.00 | L1 |
| 179 | Viscidulin I | 299.2 | 303.0498 | C₁₅H₁₀O₇ | I | P | 3.90 | 0.97 | 0.64 | 0.99 | L1 |
| 180 | Iristectorin B | 299.4 | 493.1335 | C₂₃H₂₄O₁₂ | I | P | 3.89 | 0.93 | 0.64 | 0.99 | L1 |
| 181 | Choerospondin / Prunin | 303.0 | 433.1137 | C₂₁H₂₂O₁₀ | I | N | 3.91–3.97 | 0.95 | 0.68–0.94 | 0.99–1.00 | UI |
| 182 | Kaempferol-3-*O*-galactoside | 303.9 | 449.1076 | C₂₁H₂₀O₁₁ | I | P/N | 3.87 | 0.92 | 0.62 | 0.96 | L1 |
| 183 | Apigenin-7-*O*-rutinoside / Rhoifolin | 304.1 | 579.1723 | C₂₇H₃₀O₁₄ | I | P | 3.83–3.85 | 0.83 | 0.57–0.59 | 0.95–0.98 | UI |
| 184 | Kaempferol 3-*O*-*β*-D-glucopyranosyl-(1→2)-*α*-L-rhamnopyranoside | 306.3 | 593.1504 | C₂₇H₃₀O₁₅ | I | N | 3.84 | 0.91 | 0.80 | 0.82 | L1 |
| 185 | Nicotiflorin | 306.7 | 595.1650 | C₂₇H₃₀O₁₅ | I | P | 3.96 | 0.92 | 0.93 | 1.00 | L1 |
| 186 | Theaflavin | 310.5 | 565.1318 | C₂₉H₂₄O₁₂ | I | P | 3.86 | 0.73 | 0.73 | 0.98 | L1 |
| 187 | Oroxin A | 310.9 | 433.1129 | C₂₁H₂₀O₁₀ | I | P | 3.99 | 0.99 | 0.99 | 1.00 | L1 |
| 188 | (2*S*)-2-(2,6-Dihydroxyphenyl)-5,7-dihydroxy-chroman-4-one | 317.6 | 287.0561 | C₁₅H₁₂O₆ | I | N | 3.99 | 1.00 | 1.00 | 0.97 | L1 |
| 189 | Juglanin | 318.4 | 419.0973 | C₂₀H₁₈O₁₀ | I | P/N | 3.90 | 0.99 | 0.81 | 0.90 | L1 |
| 190 | Theaflavin-3-gallate | 327.4 | 717.1452 | C₃₆H₂₈O₁₆ | I | P | 3.85 | 0.99 | 0.52 | 1.00 | L1 |
| 191 | 3′,4′-Dihydroxyflavone | 327.5 | 255.0654 | C₁₅H₁₀O₄ | I | P | 3.88 | 0.96 | 0.56 | 1.00 | L1 |
| 192 | Quercetin | 330.1 | 303.0499 | C₁₅H₁₀O₇ | I | P/N | 3.90 | 0.98 | 0.64 | 0.99 | L1 |
| 193 | 7,3',4'-Trihydroxyflavone / Sulfuretin | 336.5 | 271.0601 | C₁₅H₁₀O₅ | I | P/N | 3.91 | 0.99 | 0.69 | 0.97–0.98 | UI |
| 194 | Tiliroside | 342.4 | 595.1440 | C₃₀H₂₆O₁₃ | I | P/N | 3.90 | 0.92 | 0.82 | 0.93 | L1 |
| 195 | 6-Hydroxykaempferol 3,6-dimethyl ether | 355.5 | 331.0811 | C₁₇H₁₄O₇ | I | P | 3.94 | 0.96 | 0.87 | 0.97 | L1 |
| 196 | Nobiletin | 377.9 | 403.1385 | C₂₁H₂₂O₈ | I | P | 3.94 | 0.96 | 0.81 | 1.00 | L1 |
| 197 | Glycerol 3-phosphate | 40.0 | 171.0063 | C₃H₉O₆P | J | N | 3.91 | 0.98 | 0.73 | 0.96 | L1 |
| 198 | Glycerophosphocholine | 44.6 | 258.1099 | C₈H₂₀NO₆P | J | P | 3.89 | 0.95 | 0.68 | 0.97 | L1 |
| 199 | PC(8:0/8:0) | 466.4 | 492.3086 | C₂₄H₄₈NO₈P | J | P | 3.98 | 0.97 | 0.96 | 0.99 | L1 |
| 200 | LPC(18:3) | 475.9 | 518.3231 | C₂₆H₄₈NO₇P | J | P | 3.93 | 0.87 | 0.89 | 0.99 | L1 |
| 201 | LPE(18:3) | 480.7 | 476.2766 | C₂₃H₄₂NO₇P | J | P | 3.91 | 0.92 | 0.84 | 0.94 | L1 |
| 202 | LPC(17:2) | 481.4 | 506.3243 | C₂₅H₄₈NO₇P | J | P | 3.90 | 0.98 | 0.81 | 0.91 | L1 |
| 203 | LPC(16:1) | 487.7 | 494.3239 | C₂₄H₄₈NO₇P | J | P | 3.93 | 0.97 | 0.78 | 0.99 | L1 |
| 204 | LPC(18:2/0:0) | 495.9 | 520.3388 | C₂₆H₅₀NO₇P | J | P | 3.87 | 0.87 | 0.62 | 0.99 | L1 |
| 205 | LPC(15:0) | 497.6 | 482.3240 | C₂₃H₄₈NO₇P | J | P | 3.89 | 0.97 | 0.62 | 0.98 | L1 |
| 206 | LPC(17:1) | 504.5 | 508.3397 | C₂₅H₅₀NO₇P | J | P | 3.87 | 0.99 | 0.67 | 0.90 | L1 |
| 207 | LPC(18:1) | 512.3 | 522.3553 | C₂₆H₅₂NO₇P | J | P | 3.92 | 0.99 | 0.73 | 0.98 | L1 |
| 208 | LPC(16:0) | 513.9 | 496.3391 | C₂₄H₅₀NO₇P | J | P | 3.85 | 0.91 | 0.54 | 0.98 | L1 |
| 209 | LPE(16:0) | 518.7 | 454.2924 | C₂₁H₄₄NO₇P | J | P/N | 3.91 | 0.94 | 0.74 | 0.98 | L1 |
| 210 | LPE(18:1(9*Z*)/0:0) | 522.5 | 480.3082 | C₂₃H₄₆NO₇P | J | P/N | 3.92 | 0.98 | 0.75 | 0.98 | L1 |
| 211 | PC(10:0/10:0) | 523.5 | 548.3709 | C₂₈H₅₆NO₈P | J | P | 3.91 | 0.98 | 0.66 | 0.99 | L1 |
| 212 | PC(32:1) | 536.2 | 732.5516 | C₄₀H₇₈NO₈P | J | P | 3.95 | 0.80 | 0.99 | 1.00 | L1 |
| 213 | PC(34:2) | 551.7 | 758.5684 | C₄₂H₈₀NO₈P | J | P | 3.95 | 0.91 | 0.89 | 1.00 | L1 |
| 214 | PC(36:3) | 552.2 | 784.5844 | C₄₄H₈₂NO₈P | J | P | 3.96 | 0.94 | 0.91 | 1.00 | L1 |
| 215 | PC(38:4) | 568.1 | 810.6033 | C₄₆H₈₄NO₈P | J | P | 3.91 | 0.79 | 0.85 | 1.00 | L1 |
| 216 | PC(42:11) | 575.3 | 834.5393 | C₅₀H₇₈NO₈P | J | P | 3.88 | 0.69 | 0.82 | 1.00 | L1 |
| 217 | PC(34:5) | 589.0 | 752.5224 | C₄₂H₇₄NO₈P | J | P | 3.92 | 0.99 | 0.68 | 1.00 | L1 |
| 218 | PC(36:6) | 590.4 | 778.5366 | C₄₄H₇₆NO₈P | J | P | 3.92 | 0.87 | 0.83 | 1.00 | L1 |
| 219 | PC(37:5) | 593.1 | 794.5681 | C₄₅H₈₀NO₈P | J | P | 3.87 | 0.89 | 0.62 | 1.00 | L1 |
| 220 | PC(34:3) | 599.9 | 756.5521 | C₄₂H₇₈NO₈P | J | P | 3.93 | 0.85 | 0.88 | 1.00 | L1 |
| 221 | PC(35:5) | 600.5 | 766.5378 | C₄₃H₇₆NO₈P | J | P | 3.89 | 0.97 | 0.60 | 1.00 | L1 |
| 222 | PC(34:4) | 606.6 | 754.5377 | C₄₂H₇₆NO₈P | J | P | 3.88 | 0.97 | 0.54 | 1.00 | L1 |
| 223 | PC(36:5) | 607.3 | 780.5521 | C₄₄H₇₈NO₈P | J | P | 3.88 | 0.85 | 0.67 | 1.00 | L1 |
| 224 | PC(38:6) | 614.7 | 806.5692 | C₄₆H₈₀NO₈P | J | P | 3.99 | 0.97 | 0.97 | 1.00 | L1 |
| 225 | PC(16:0/20:4) | 622.4 | 782.5675 | C₄₄H₈₀NO₈P | J | P | 3.91 | 0.83 | 0.81 | 1.00 | L1 |
| 226 | PG(18:1/18:1(9*Z*)) | 634.5 | 773.5331 | C₄₂H₇₉O₁₀P | J | N | 3.92 | 0.94 | 0.83 | 0.95 | L1 |
| 227 | PG(16:0/18:1) | 636.5 | 747.5169 | C₄₀H₇₇O₁₀P | J | N | 3.89 | 0.89 | 0.91 | 0.88 | L1 |
| 228 | PC(38:5) | 638.5 | 808.5850 | C₄₆H₈₂NO₈P | J | P | 3.95 | 0.99 | 0.79 | 1.00 | L1 |
| 229 | PE(34:2) | 641.9 | 716.5216 | C₃₉H₇₄NO₈P | J | P | 3.90 | 0.91 | 0.69 | 0.99 | L1 |
| 230 | Tachioside | 99.7 | 301.0928 | C₁₃H₁₈O₈ | K | N | 3.87 | 0.98 | 0.52 | 1.00 | L1 |
| 231 | Dianthoside | 130.3 | 289.0920 | C₁₂H₁₆O₈ | K | P | 3.98 | 0.95 | 0.98 | 0.99 | L1 |
| 232 | Sibiricose A3 | 213.6 | 461.1295 | C₁₉H₂₆O₁₃ | K | N | 3.92 | 0.92 | 0.87 | 0.94 | L1 |
| 233 | 1-Salicylate glucuronide | 221.0 | 295.0459 | C₁₃H₁₄O₉ | K | N | 3.87 | 1.00 | 0.86 | 0.81 | L1 |
| 234 | Salidroside | 227.0 | 299.1136 | C₁₄H₂₀O₇ | K | N | 3.96 | 0.99 | 0.96 | 0.95 | L1 |
| 235 | 3-Methyladenine | 52.1 | 150.0772 | C₆H₇N₅ | L | P | 3.86 | 0.91 | 0.55 | 1.00 | L1 |
| 236 | Uridine diphosphate | 58.1 | 402.9947 | C₉H₁₄N₂O₁₂P₂ | L | N | 3.96 | 0.97 | 0.97 | 0.95 | L1 |
| 237 | Guanosine monophosphate | 61.3 | 362.0505 | C₁₀H₁₄N₅O₈P | L | N | 3.88 | 0.96 | 0.66 | 0.95 | L1 |
| 238 | Guanine | 68.0 | 152.0566 | C₅H₅N₅O | L | P | 3.90 | 0.93 | 0.66 | 1.00 | L1 |
| 239 | 2-Aminopurine | 72.7 | 136.0617 | C₅H₅N₅ | L | P | 3.92 | 0.95 | 0.75 | 1.00 | L1 |
| 240 | Adenine | 74.3 | 136.0617 | C₅H₅N₅ | L | P/N | 3.89 | 0.96 | 0.60 | 1.00 | L1 |
| 241 | Uridine | 78.9 | 243.0621 | C₉H₁₂N₂O₆ | L | N | 3.82 | 0.97 | 0.50 | 0.91 | L1 |
| 242 | Adenosine monophosphate | 80.7 | 348.0702 | C₁₀H₁₄N₅O₇P | L | P/N | 3.93 | 0.94 | 0.84 | 0.97 | L1 |
| 243 | Thymine | 80.8 | 127.0501 | C₅H₆N₂O₂ | L | P | 3.98 | 0.95 | 0.99 | 1.00 | L1 |
| 244 | 1-Methylguanine / 7-Methylguanine | 87.7 | 166.0723 | C₆H₇N₅O | L | P | 3.96–3.97 | 0.97 | 0.86–0.90 | 1.00 | UI |
| 245 | *S*-Adenosyl-L-homocysteine | 94.9 | 385.1290 | C₁₄H₂₀N₆O₅S | L | P | 3.95 | 0.98 | 0.82 | 0.99 | L1 |
| 246 | 2′-Deoxyguanosine 5′-monophosphate | 95.1 | 346.0548 | C₁₀H₁₄N₅O₇P | L | N | 3.92 | 0.81 | 0.92 | 0.97 | L1 |
| 247 | Guanosine | 96.1 | 284.0990 | C₁₀H₁₃N₅O₅ | L | P/N | 3.92 | 0.98 | 0.72 | 0.99 | L1 |
| 248 | Deoxyguanosine | 115.4 | 266.0896 | C₁₀H₁₃N₅O₄ | L | N | 3.88 | 0.95 | 0.69 | 0.94 | L1 |
| 249 | Xanthosine | 115.5 | 285.0831 | C₁₀H₁₂N₄O₆ | L | P/N | 3.95 | 0.97 | 0.88 | 0.98 | L1 |
| 250 | Adenosine 3'-monophosphate | 153.4 | 348.0707 | C₁₀H₁₄N₅O₇P | L | P/N | 3.88 | 0.96 | 0.60 | 0.98 | L1 |
| 251 | 1-Methylguanosine | 163.4 | 298.1147 | C₁₁H₁₅N₅O₅ | L | P | 3.98 | 0.98 | 0.92 | 1.00 | L1 |
| 252 | 2'-*O*-Methylguanosine | 168.0 | 298.1147 | C₁₁H₁₅N₅O₅ | L | P/N | 3.94 | 0.98 | 0.81 | 0.98 | L1 |
| 253 | Adenosine | 186.3 | 268.1036 | C₁₀H₁₃N₅O₄ | L | P/N | 3.93 | 0.93 | 0.82 | 0.98 | L1 |
| 254 | 2-Methylguanosine | 194.7 | 298.1148 | C₁₁H₁₅N₅O₅ | L | P | 3.91 | 0.96 | 0.66 | 1.00 | L1 |
| 255 | 5'-Deoxyadenosine / Deoxyadenosine | 201.0 | 252.1092 | C₁₀H₁₃N₅O₃ | L | P | 3.89–3.97 | 0.99 | 0.56–0.88 | 1.00 | UI |
| 256 | Succinyladenosine | 209.8 | 382.1001 | C₁₄H₁₇N₅O₈ | L | N | 3.89 | 0.95 | 0.74 | 0.93 | L1 |
| 257 | 3'-*O*-Methyladenosine | 215.5 | 282.1197 | C₁₁H₁₅N₅O₄ | L | P | 3.88 | 0.99 | 0.55 | 1.00 | L1 |
| 258 | Malic acid | 46.1 | 133.0141 | C₄H₆O₅ | M | N | 3.92 | 0.94 | 0.77 | 0.98 | L1 |
| 259 | Quinic acid | 46.1 | 173.0455 | C₇H₁₂O₆ | M | N | 3.95 | 0.99 | 0.97 | 0.92 | L1 |
| 260 | Isocitric acid | 46.7 | 191.0195 | C₆H₈O₇ | M | N | 3.86 | 0.93 | 0.82 | 0.84 | L1 |
| 261 | Citric Acid | 57.9 | 191.0195 | C₆H₈O₇ | M | N | 3.93 | 0.94 | 0.80 | 0.99 | L1 |
| 262 | Pyruvate | 60.7 | 87.0087 | C₃H₄O₃ | M | N | 3.95 | 0.93 | 0.87 | 1.00 | L1 |
| 263 | Homocitric acid | 61.4 | 205.0353 | C₇H₁₀O₇ | M | N | 3.84 | 0.98 | 0.72 | 0.83 | L1 |
| 264 | Itaconic acid | 91.9 | 129.0193 | C₅H₆O₄ | M | N | 3.90 | 0.99 | 0.61 | 1.00 | L1 |
| 265 | 2-Furoic acid | 93.8 | 111.0087 | C₅H₄O₃ | M | N | 3.84 | 0.95 | 0.75 | 0.82 | L1 |
| 266 | Pyrogallol | 94.7 | 127.0389 | C₆H₆O₃ | N | P/N | 3.92 | 0.97 | 0.70 | 1.00 | L1 |
| 267 | Hydroquinone / Pyrocatechol | 225.5 | 109.0294 | C₆H₆O₂ | N | N | 3.89 | 0.96 | 0.59–0.60 | 0.99–1.00 | UI |
| 268 | 4-Hydroxybenzaldehyde | 263.9 | 123.0437 | C₇H₆O₂ | N | P/N | 3.88 | 0.88 | 0.74 | 0.95 | L1 |
| 269 | 3-Hydroxybenzaldehyde | 270.0 | 121.0294 | C₇H₆O₂ | N | N | 3.98 | 0.95 | 0.97 | 1.00 | L1 |
| 270 | Phenol | 277.2 | 93.0345 | C₆H₆O | N | N | 3.95 | 0.95 | 0.86 | 1.00 | L1 |
| 271 | 2',6'-Dihydroxyacetophenone | 325.6 | 151.0400 | C₈H₈O₃ | N | N | 3.87 | 0.99 | 0.64 | 0.93 | L1 |
| 272 | 4-Vinylphenol | 366.9 | 119.0502 | C₈H₈O | N | N | 3.92 | 0.97 | 0.70 | 1.00 | L1 |
| 273 | 2'-Hydroxy-5'-methylacetophenone | 390.5 | 149.0608 | C₉H₁₀O₂ | N | N | 3.90 | 0.99 | 0.66 | 0.98 | L1 |
| 274 | 2-Hydroxycinnamic acid | 221.3 | 163.0400 | C₉H₈O₃ | O | N | 3.96 | 0.97 | 0.86 | 1.00 | L1 |
| 275 | 5-*O*-Coumaroylquinic acid | 230.1 | 339.1073 | C₁₆H₁₈O₈ | O | P/N | 3.81 | 0.94 | 0.61 | 0.85 | L1 |
| 276 | Chlorogenic acid | 235.4 | 355.1021 | C₁₆H₁₈O₉ | O | P/N | 3.92 | 0.92 | 0.76 | 1.00 | L1 |
| 277 | Caffeic acid | 235.9 | 181.0494 | C₉H₈O₄ | O | P/N | 3.89 | 0.95 | 0.69 | 0.96 | L1 |
| 278 | Scopolin | 236.8 | 353.0872 | C₁₆H₁₈O₉ | O | N | 3.80 | 0.89 | 0.63 | 0.85 | L1 |
| 279 | 4-*p*-Coumaroylquinic acid | 253.4 | 339.1072 | C₁₆H₁₈O₈ | O | P/N | 3.87 | 0.91 | 0.65 | 0.97 | L1 |
| 280 | Esculetin | 255.1 | 177.0192 | C₉H₆O₄ | O | N | 3.88 | 0.97 | 0.62 | 0.97 | L1 |
| 281 | 4-*O*-Feruloyl-D-quinic acid | 255.3 | 367.1031 | C₁₇H₂₀O₉ | O | N | 3.90 | 0.94 | 0.68 | 1.00 | L1 |
| 282 | Fraxinol | 275.8 | 223.0599 | C₁₁H₁₀O₅ | O | P | 3.96 | 0.93 | 0.99 | 0.96 | L1 |
| 283 | Coniferaldehyde | 281.8 | 179.0702 | C₁₀H₁₀O₃ | O | P | 3.80 | 0.95 | 0.63 | 0.82 | L1 |
| 284 | Microminutin | 282.7 | 273.0755 | C₁₅H₁₂O₅ | O | P | 3.82 | 0.94 | 0.67 | 0.84 | L1 |
| 285 | Methyl caffeate | 287.2 | 193.0505 | C₁₀H₁₀O₄ | O | N | 3.82 | 0.97 | 0.63 | 0.83 | L1 |
| 286 | 6-Hydroxy-4-methylcoumarin | 287.7 | 177.0544 | C₁₀H₈O₃ | O | P | 3.92 | 0.91 | 0.82 | 0.97 | L1 |
| 287 | 3-(2,4-Dihydroxypentyl)-6,8-dihydroxyisochromen-1-one | 288.5 | 279.0871 | C₁₄H₁₆O₆ | O | N | 3.91 | 0.93 | 0.87 | 0.91 | L1 |
| 288 | Umbelliferone | 288.5 | 161.0243 | C₉H₆O₃ | O | N | 3.93 | 0.96 | 0.79 | 0.98 | L1 |
| 289 | 4-Hydroxycinnamic acid | 289.0 | 165.0545 | C₉H₈O₃ | O | P/N | 3.88 | 0.95 | 0.62 | 0.98 | L1 |
| 290 | 5-*O*-Cinnamoylquinic acid | 294.9 | 323.1124 | C₁₆H₁₈O₇ | O | P | 3.87 | 0.97 | 0.53 | 1.00 | L1 |
| 291 | 1,4-Dicaffeoylquinic acid / 4,5-Dicaffeoylquinic acid | 299.6 | 517.1338 | C₂₅H₂₄O₁₂ | O | P/N | 3.82–3.83 | 0.90 | 0.56–0.64 | 0.90–0.91 | UI |
| 292 | 3-Hydroxycinnamic acid | 323.6 | 163.0400 | C₉H₈O₃ | O | N | 3.91 | 0.99 | 0.79 | 0.93 | L1 |
| 293 | 7-Methoxycoumarin | 334.6 | 177.0545 | C₁₀H₈O₃ | O | P | 3.88 | 0.93 | 0.72 | 0.94 | L1 |
| 294 | Demethoxycapillarisin | 348.6 | 287.0549 | C₁₅H₁₀O₆ | O | P | 3.87 | 0.96 | 0.52 | 1.00 | L1 |
| 295 | 4-Methoxycinnamic acid | 350.7 | 177.0557 | C₁₀H₁₀O₃ | O | N | 3.83 | 0.99 | 0.68 | 0.82 | L1 |
| 296 | 8-Geranyl-7-hydroxycoumarin | 460.1 | 297.1496 | C₁₉H₂₂O₃ | O | N | 3.97 | 0.99 | 0.90 | 0.99 | L1 |
| 297 | Phloroglucinol | 84.8 | 127.0389 | C₆H₆O₃ | P | P/N | 3.91 | 0.95 | 0.73 | 0.98 | L1 |
| 298 | 2,4,6-Trihydroxybenzaldehyde | 294.6 | 153.0192 | C₇H₆O₄ | P | N | 3.92 | 0.97 | 0.74 | 0.99 | L1 |
| 299 | 2',4',6'-Trihydroxyacetophenone | 316.7 | 167.0349 | C₈H₈O₄ | P | N | 3.91 | 0.96 | 0.74 | 0.96 | L1 |
| 300 | Aurantio-obtusin *β*-D-glucoside | 316.6 | 493.1344 | C₂₃H₂₄O₁₂ | Q | P | 3.91 | 0.96 | 0.70 | 0.98 | L1 |
| 301 | 1,3,5,6-Tetrahydroxy-2-methylanthraquinone | 411.5 | 285.0404 | C₁₅H₁₀O₆ | Q | N | 3.94 | 0.99 | 0.79 | 1.00 | L1 |
| 302 | Icariside C1 | 334.8 | 417.2492 | C₂₁H₃₈O₈ | R | N | 3.94 | 0.98 | 0.86 | 0.97 | L1 |
| 303 | Acuminoside / Kenposide A / Geranyl *β*-primeveroside | 362.3 | 447.2231 | C₂₁H₃₆O₁₀ | R | N | 3.85–3.87 | 0.93 | 0.50–0.56 | 0.98–0.99 | UI |
| 304 | Arjungenin / Polygalacic acid / Sericic acid / Terminolic acid | 405.1 | 503.3376 | C₃₀H₄₈O₆ | R | N | 3.84–3.94 | 0.97 | 0.57–0.78 | 0.90–1.00 | UI |
| 305 | Sanguic acid | 410.0 | 517.3172 | C₃₀H₄₆O₇ | R | N | 3.89 | 0.99 | 0.56 | 1.00 | L1 |
| 306 | Cecropiacic acid | 423.7 | 517.3172 | C₃₀H₄₆O₇ | R | N | 3.87 | 0.99 | 0.52 | 0.99 | L1 |
| 307 | Ethyl chrysanthemate | 426.3 | 195.1390 | C₁₂H₂₀O₂ | R | N | 3.94 | 0.99 | 0.79 | 1.00 | L1 |
| 308 | 1-Deoxypebrolide | 429.2 | 415.2113 | C₂₄H₃₀O₆ | R | P | 3.96 | 0.96 | 0.90 | 1.00 | L1 |
| 309 | (+)-Cyperotundone / *α*-Cyperone | 465.0 | 219.1742 | C₁₅H₂₂O | R | P | 3.88–3.91 | 0.95 | 0.59–0.91 | 0.90–1.00 | UI |
| 310 | Orthosphenic acid | 527.7 | 487.3428 | C₃₀H₄₈O₅ | R | N | 3.96 | 0.99 | 0.91 | 0.97 | L1 |
| 311 | Nicotinate | 60.8 | 124.0393 | C₆H₅NO₂ | S | P/N | 3.97 | 0.98 | 0.93 | 0.99 | L1 |
| 312 | Pantothenic acid | 166.6 | 220.1180 | C₉H₁₇NO₅ | S | P/N | 3.89 | 0.99 | 0.66 | 0.96 | L1 |
| 313 | Folinic acid | 210.5 | 472.1585 | C₂₀H₂₃N₇O₇ | S | N | 3.88 | 0.99 | 0.74 | 0.90 | L1 |
| 314 | *myo*-Inositol | 43.4 | 179.0560 | C₆H₁₂O₆ | T | N | 3.88 | 0.96 | 0.67 | 0.94 | L1 |
| 315 | Glycolate | 44.1 | 75.0087 | C₂H₄O₃ | T | N | 3.96 | 0.95 | 0.90 | 1.00 | L1 |
| 316 | Glyoxylic acid | 46.2 | 72.9931 | C₂H₂O₃ | T | N | 3.97 | 0.97 | 0.90 | 1.00 | L1 |
| 317 | Gulono-1,4-lactone | 46.2 | 177.0404 | C₆H₁₀O₆ | T | N | 3.82 | 0.98 | 0.64 | 0.83 | L1 |
| 318 | Hydroxyacetone | 62.7 | 73.0294 | C₃H₆O₂ | T | N | 3.90 | 0.97 | 0.65 | 1.00 | L1 |
| 319 | 5-Hydroxymethylfurfural | 117.5 | 127.0390 | C₆H₆O₃ | T | P/N | 3.83 | 0.98 | 0.55 | 0.90 | L1 |
| 320 | 2-Methylcyclopentane-1,3-dione | 164.0 | 113.0597 | C₆H₈O₂ | T | P | 3.88 | 0.97 | 0.59 | 0.97 | L1 |
| 321 | 3,3-Dimethylcyclohexanone | 322.0 | 109.1011 | C₈H₁₄O | T | P | 3.92 | 0.97 | 0.88 | 0.92 | L1 |
| 322 | (*S*)-Abscisic acid | 334.7 | 265.1433 | C₁₅H₂₀O₄ | T | P/N | 3.83 | 0.98 | 0.53 | 0.90 | L1 |
| 323 | Isophorone | 356.8 | 139.1116 | C₉H₁₄O | T | P | 3.92 | 0.94 | 0.76 | 1.00 | L1 |
| 324 | Jasmone | 409.3 | 165.1273 | C₁₁H₁₆O | T | P | 3.97 | 0.93 | 0.97 | 0.98 | L1 |
| 325 | Anisylacetone | 429.1 | 177.0920 | C₁₁H₁₄O₂ | T | N | 3.95 | 0.99 | 0.83 | 1.00 | L1 |
| 326 | Asperphenamate | 446.4 | 507.2275 | C₃₂H₃₀N₂O₄ | T | P | 3.97 | 0.96 | 0.97 | 0.97 | L1 |
| 327 | Iraldeine | 465.2 | 193.1586 | C₁₃H₂₀O | T | P | 3.91 | 0.95 | 0.79 | 0.95 | L1 |
| 328 | SM(d34:1) | 577.7 | 685.5655 | C₃₉H₇₉N₂O₆P | T | P | 3.89 | 0.89 | 0.70 | 1.00 | L1 |

*ᵃ* RT, retention time.
*ᵇ* Class codes: A, Alkaloids; B, Amides; C, Amino acids and peptides; D, Aromatic acids; E, Carbohydrates; F, Chromones; G, Esters; H, Fatty acids and derivatives; I, Flavonoids; J, Glycerophospholipids; K, Glycosides; L, Nucleotides and derivatives; M, Organic acids and derivatives; N, Phenols; O, Phenylpropanoids; P, Phloroglucinols; Q, Quinones; R, Terpenoids; S, Vitamins; T, Others.
*ᶜ* P, positive ion mode; N, negative ion mode; P/N, detected in both positive and negative ion modes.
*ᵈ* *S*_total_, total identification score.
*ᵉ* *S*_m/z_, precursor *m/z* match score.
*ᶠ S*_RT_, retention-time match score.
*ᵍ* *S*_MS/MS_, MS/MS spectral match score.
*ʰ* IS, identification status; L1, Level 1 identification based on matching with authentic standards in the in-house library; UI, unresolved isomeric feature.

**Table S3.** The quality parameters of PLS-DA models in Fig. 3.

| PLS-DA plots | R^2^X (cum) | R^2^Y (cum) | Q^2^ (cum) |
| --- | --- | --- | --- |
| Fig. 3D | 0.906 | 0.996 | 0.990 |
| Fig. 3E | 0.941 | 0.994 | 0.985 |
| Fig. 3F | 0.838 | 0.999 | 0.990 |

**Table S4.** Spearman correlations between grade-associated non-volatile features and antioxidant indices of Gougunao tea.

| Ion mode | No.*^a^* | Compound | DPPH | | ABTS | | FRAP | |
| --- | --- | --- | --- | --- | --- | --- | --- | --- |
|  |  |  | ρ*^b^* | *p value^c^* | ρ | *p value* | ρ | *p value* |
| Positive | 9 | Theophylline | -0.371 | 0.468 | -0.314 | 0.544 | 0.029 | 0.957 |
| Positive | 12 | Caffeine | -0.886 | 0.019* | -0.829 | 0.042* | -0.086 | 0.872 |
| Positive | 18 | Linoleamide | 0.657 | 0.156 | 0.600 | 0.208 | 0.086 | 0.872 |
| Positive | 42 | Theanine | -0.029 | 0.957 | 0.086 | 0.872 | 0.429 | 0.397 |
| Positive | 47 | Leucine | -0.600 | 0.208 | -0.657 | 0.156 | -0.543 | 0.266 |
| Positive | 50 | Phenylalanine | 0.029 | 0.957 | -0.086 | 0.872 | -0.429 | 0.397 |
| Positive | 53 | Tryptophan | -0.086 | 0.872 | -0.143 | 0.787 | -0.200 | 0.704 |
| Positive | 155 | (+)-Gallocatechin | 0.371 | 0.468 | 0.257 | 0.623 | 0.086 | 0.872 |
| Positive | 157 | Epigallocatechin | 0.486 | 0.329 | 0.314 | 0.544 | -0.200 | 0.704 |
| Positive | 159 | Procyanidin B2 | -0.257 | 0.623 | -0.086 | 0.872 | 0.486 | 0.329 |
| Positive | 164 | Epigallocatechin gallate / Gallocatechin gallate | 0.029 | 0.957 | 0.086 | 0.872 | 0.314 | 0.544 |
| Positive | 165 | Corymboside / Isoschaftoside / Schaftoside / Vicenin 3 | 0.257 | 0.623 | 0.143 | 0.787 | -0.143 | 0.787 |
| Positive | 171 | (-)-Catechin gallate | 0.029 | 0.957 | 0.257 | 0.623 | 0.771 | 0.072 |
| Positive | 176 | Rutin | 0.429 | 0.397 | 0.257 | 0.623 | 0.029 | 0.957 |
| Positive | 185 | Nicotiflorin | 0.429 | 0.397 | 0.200 | 0.704 | -0.257 | 0.623 |
| Positive | 204 | LPC(18:2/0:0) | -0.029 | 0.957 | -0.143 | 0.787 | -0.257 | 0.623 |
| Positive | 208 | LPC(16:0) | 0.371 | 0.468 | 0.200 | 0.704 | -0.600 | 0.208 |
| Positive | 218 | PC(36:6) | 0.200 | 0.704 | 0.371 | 0.468 | 0.600 | 0.208 |
| Positive | 223 | PC(36:5) | 0.086 | 0.872 | 0.257 | 0.623 | 0.486 | 0.329 |
| Positive | 253 | Adenosine | 0.086 | 0.872 | 0.029 | 0.957 | -0.143 | 0.787 |
| Positive | 308 | 1-Deoxypebrolide | 0.600 | 0.208 | 0.714 | 0.111 | 0.486 | 0.329 |
| Negative | 39 | Pyroglutamic acid | -0.086 | 0.872 | -0.257 | 0.623 | -0.314 | 0.544 |
| Negative | 42 | Theanine | -0.029 | 0.957 | 0.086 | 0.872 | 0.429 | 0.397 |
| Negative | 47 | Leucine | -0.600 | 0.208 | -0.657 | 0.156 | -0.543 | 0.266 |
| Negative | 53 | Tryptophan | -0.086 | 0.872 | -0.143 | 0.787 | -0.200 | 0.704 |
| Negative | 54 | *N*-Acetyltryptophan | 0.143 | 0.787 | -0.029 | 0.957 | -0.200 | 0.704 |
| Negative | 58 | Corilagin | -0.257 | 0.623 | -0.086 | 0.872 | 0.543 | 0.266 |
| Negative | 60 | Methylgallate | -0.371 | 0.468 | -0.257 | 0.623 | -0.086 | 0.872 |
| Negative | 75 | Isomaltose / Gentiobiose / Trehalose / Palatinose / Galactinol / Neotrehalose / Turanose / Sucrose | -0.143 | 0.787 | -0.200 | 0.704 | 0.257 | 0.623 |
| Negative | 135 | 12-Hydroxystearic acid | 0.543 | 0.266 | 0.429 | 0.397 | -0.371 | 0.468 |
| Negative | 139 | *α*-Linolenic acid | 0.086 | 0.872 | 0.143 | 0.787 | 0.200 | 0.704 |
| Negative | 155 | (+)-Gallocatechin | 0.371 | 0.468 | 0.257 | 0.623 | 0.086 | 0.872 |
| Negative | 157 | Epigallocatechin | 0.486 | 0.329 | 0.314 | 0.544 | -0.200 | 0.704 |
| Negative | 158 | Leucocyanidin | 0.486 | 0.329 | 0.314 | 0.544 | -0.200 | 0.704 |
| Negative | 159 | Procyanidin B2 | -0.257 | 0.623 | -0.086 | 0.872 | 0.486 | 0.329 |
| Negative | 175 | Quercetin-3′-*β*-D-glucopyranosyl-(1→2)-*α*-L-rhamnopyranoside | 0.429 | 0.397 | 0.257 | 0.623 | 0.029 | 0.957 |
| Negative | 176 | Rutin | 0.429 | 0.397 | 0.257 | 0.623 | 0.029 | 0.957 |
| Negative | 182 | Kaempferol-3-*O*-galactoside | 0.600 | 0.208 | 0.486 | 0.329 | -0.086 | 0.872 |
| Negative | 184 | Kaempferol 3-*O*-*β*-D-glucopyranosyl-(1→2)-*α*-L-rhamnopyranoside | 0.429 | 0.397 | 0.200 | 0.704 | -0.257 | 0.623 |
| Negative | 192 | Quercetin | 0.257 | 0.623 | 0.143 | 0.787 | -0.143 | 0.787 |
| Negative | 194 | Tiliroside | -0.029 | 0.957 | 0.086 | 0.872 | 0.429 | 0.397 |
| Negative | 258 | Malic acid | -0.200 | 0.704 | -0.029 | 0.957 | 0.600 | 0.208 |
| Negative | 260 | Isocitric acid | -0.086 | 0.872 | 0.029 | 0.957 | 0.314 | 0.544 |
| Negative | 261 | Citric Acid | -0.200 | 0.704 | -0.029 | 0.957 | 0.600 | 0.208 |
| Negative | 275 | 5-*O*-Coumaroylquinic acid | 0.257 | 0.623 | 0.086 | 0.872 | -0.543 | 0.266 |
| Negative | 276 | Chlorogenic acid | -0.371 | 0.468 | -0.257 | 0.623 | 0.029 | 0.957 |
| Negative | 278 | Scopolin | 0.143 | 0.787 | -0.086 | 0.872 | -0.314 | 0.544 |
| Negative | 279 | 4-*p*-Coumaroylquinic acid | -0.200 | 0.704 | -0.371 | 0.468 | -0.600 | 0.208 |
| Negative | 287 | 3-(2,4-Dihydroxypentyl)-6,8-dihydroxyisochromen-1-one | 0.257 | 0.623 | 0.371 | 0.468 | 0.257 | 0.623 |
| Negative | 303 | Acuminoside / Kenposide A / Geranyl *β*-primeveroside | -0.486 | 0.329 | -0.600 | 0.208 | -0.486 | 0.329 |

*^a^* Feature numbers correspond to those listed in Table S2.

*^b^* Spearman’s rank correlation coefficient; correlation analysis was based on six grade-level observations (*n* = 6).

*^c^* Two-tailed *p* values; * *p* < 0.05.

**Fig. S1.** DPPH and ABTS radical-scavenging activities of GT samples at a test concentration of 50 μg/mL. LAA, L-ascorbic acid. G1-G6 represent Premium Special Grade, Tribute Special Grade, Supreme Special Grade, Special Grade, Grade I, and Standard Grade, respectively.
